# General two-parameter distribution: Statistical properties, estimation, and application on COVID-19

**DOI:** 10.1371/journal.pone.0281474

**Published:** 2023-02-08

**Authors:** Ahmed M. Gemeay, Zeghdoudi Halim, M. M. Abd El-Raouf, Eslam Hussam, Alanazi Talal Abdulrahman, Nour Khaled Mashaqbah, Nawaf Alshammari, Nicholas Makumi

**Affiliations:** 1 Department of Mathematics, Faculty of Science, Tanta University, Tanta, Egypt; 2 LaPS laboratory, Badji mokhtar University, Annaba, Algeria; 3 Basic and Applied Science Institute, Arab Academy for Science, Technology and Maritime Transport (AASTMT), Alexandria, Egypt; 4 Department of Mathematics, Faculty of Science, Helwan University, Cairo, Egypt; 5 Department of Mathematics, College of Science, University of Ha’il, Ha’il, Saudi Arabia; 6 Department of educational administration, Faculty of Education, University of Ha’il, Ha’il, Saudi Arabia; 7 Biology Department, College of Science, University of Ha’il, Ha’il, Saudi Arabia; 8 Pan African University, Institute for Basic Sciences, Technology and Innovation (PAUSTI), Nairobi, Kenya; University of Bradford, UNITED KINGDOM

## Abstract

In this paper, we introduced a novel general two-parameter statistical distribution which can be presented as a mix of both exponential and gamma distributions. Some statistical properties of the general model were derived mathematically. Many estimation methods studied the estimation of the proposed model parameters. A new statistical model was presented as a particular case of the general two-parameter model, which is used to study the performance of the different estimation methods with the randomly generated data sets. Finally, the COVID-19 data set was used to show the superiority of the particular case for fitting real-world data sets over other compared well-known models.

## 1 Introduction

The spread of COVID-19 has caused international harm and economic instability in recent months. Scientists are looking into this event in great detail right now. But it’s essential to have the right facts and numbers in order to do everything you can to stop COVID-19. In the study and use of big data sciences, it is always important to give the best possible description of the data being looked at. Recent research has shown how statistical distributions can be used to model data in applied sciences, especially in medical science. Statisticians often explore new statistical models to suit data sets in diverse domains. Statistical models are very useful in describing and predicting real phenomena. Many distributions have been widely used for data modeling in several domains during the last decades. Recent developments focus on defining new families that extend well-known distributions and, at the same time, provide great flexibility in data modeling in practice. Thus, several distributions used to model lifetime data have been proposed in the statistical literature.

Academics have a better understanding of the spread of viruses and how it affects people due to their work modeling pandemics. Most nations’ adoption of “strict” health and safety safeguards has slowed down the rate at which COVID-19 spreads worldwide. Many researchers studied this pandemic such as Nagy et al. [1], Hossam et al. [2], Khan et al. [3], Abu El Azm et al. [4], Riad et al. [5], Sindhu et al. [6], Meriem et al. [7], Hassan et al. [8], Akgül et al. [9], Alsuhabi et al. [10], Almetwally [11], Caccavo [12] and Liu et al. [13].

Statistical models can be used to describe and predict real-world events. In recent years, a variety of distributions have been employed for data modeling in a variety of domains. Recent advances have centered on establishing new families that extend well-known distributions while allowing a great deal of flexibility in data modeling in practice. Several distributions have been proposed in the statistical literature to modify lifetime data, including the Lindley, XLindley [14], exponential, Zeghdoudi [15], and Xgamma [16] distributions.

Due to the existence of a single parameter, Lindley, XLindley, and Xgamma distributions need to provide more flexibility to analyze different types of lifetime data. To increase flexibility for modeling purposes, it will be useful to consider other alternatives for this distribution.

This work investigates a new polynomial exponential family that includes the distributions of XLindley and Xgamma, as well as Lindley as special instances, to introduce a new family of two-parameter continuous distributions. The existing literature on modeling survival data, biological sciences, and actuarial sciences will benefit from this new family of distributions.

This paper is organized as follows. In Section 2, we explain the formulation of the new proposed model. Section 3 contains different statistical properties of the proposed model, such as moments, incomplete moments, entropy, and stochastic orders. Also, behavior and possibles shapes for both the probability density function (PDF) and hazard rate function (HRF) are presented in Section 3. Determining unknown parameters of the proposed model by using different estimation methods was presented in 4. A special case of the proposed model was introduced in Section 5. This special case was used to check the behavior of the different estimation methods in Section 6. In Section 7, the COVID-19 data set was used to show the flexibility of the proposed model by using its special case.

## 2 Formulation of general two-parameter distribution (GTPD)

Let *X* be a random variable following the two-parameter distribution called GTPD with PDF defined as follows

$$\begin{array}{c} f(x;\theta ,k)=h(\theta ,k)p(x,\theta ,k)exp(-\theta x), \end{array}$$ (1)

where $h\left(\theta ,k\right)=\frac{\theta^{k+1}}{\theta^{k}b\left(\theta \right)+k!}$ and *p*(*x*, *θ*, *k*) = *b*(*θ*) + *x*^*k*^. We can note immediately that *f*(*x*; *θ*, *k*) is non-negative for *x*, *θ* > 0, *k* ≥ 0 and $\int_{0}^{\infty }f\left(x;\theta ,k\right)dx=1$.

Now, we can rewrite the PDF (1) of the GTPD as follows

$$\begin{array}{cc} f(x;\theta ,k) & =\frac{\theta^{k+1}}{\theta^{k}b\left(\theta \right)+k!}[b\left(\theta \right)+x^{k}]e^{-\theta x}\\ & =zf(x,\theta )+(1-z)g(x,\theta ,k),\;x,\;\theta ,\;k>0, \end{array}$$ (2)

where $z=\frac{\theta^{k}b\left(\theta \right)}{\theta^{k}b\left(\theta \right)+k}$, *f*(*x*; *θ*) = *θe*^−*θx*^ and $g\left(x;\theta ,k\right)=\frac{\theta^{k+1}}{k!}x^{k}e^{-\theta x}$.

Also, we can call the distribution in (2) by a novel mixture exponential gamma distribution (NMEGD). From (2), we can see that the new distribution is a two-component mixture of exponential distribution (with shape *θ*) and a gamma distribution (with shape *k* + 1 and scale *θ*), with mixing proportion $z=\frac{\theta^{k}b\left(\theta \right)}{\theta^{k}b\left(\theta \right)+k}.$

The cumulative distribution function (CDF) and HRF of GTPD are, respectively, defined as follows

$$F(x;\theta ,k)=\frac{\theta^{k}[b\left(\theta \right)e^{-\theta x}+\theta \Gamma \left(k+1,x\theta \right)]}{\theta^{k}b\left(\theta \right)+k!},$$ (3)

$$h(x;\theta ,k)=\frac{\theta (b\left(\theta \right)+x^{k})}{b\left(\theta \right)+\theta^{-k}e^{\theta x}\Gamma \left(k+1,x\theta \right)}.$$ (4)

## 3 Statistical properties

### 3.1 Behavior of PDF and HRF of GTPD

This subsection discusses the behavior and possible shapes of the PDF (2) and HRF (4). Also, we determined the mode of GTPD in this subsection.

The PDF behavior is described as follows

$$\underset{x\to 0}{\text{lim}}f\left(x\right)=\frac{\theta^{k+1}b\left(\theta \right)}{\theta^{k}b\left(\theta \right)+k!},\;\underset{x\to \infty }{\text{lim}}f\left(x\right)=0.$$

The possible shapes of PDF (2) are studied as follows

- When *k* = 1, the first derivative of PDF (2) with respect to *x* is determined as follows
  $$\frac{df(x)}{dx}=-\theta^{2}\frac{e^{-x\theta }}{\theta b(\theta )+1}(\theta b(\theta )+x\theta -1).$$
  When *θb* (*θ*) − 1 < 0, we have a critical point $x_{0}=-\frac{\theta b(\theta )-1}{\theta }$ which maximize PDF (2).
  When *θb* (*θ*) − 1 ≥ 0, we have $\frac{d}{dx}f\left(x\right)\leq 0$, then the PDF (2) is decreasing in *x*.
- When *k* ≠ 1, the first derivative of PDF (2) with respect to *x* is determined as follows
  $$\frac{df(x)}{dx}=\theta^{k+1}\frac{e^{-x\theta }}{k!+b\theta^{k}}J(x^{k-1}),$$
  where *J*(*x*^*k*−1^) = −*θb* + *kx*^*k*−1^ − *x*^*k*^*θ*.
  **Case I:** *k* < 1
  i- For *k* − *θ*(*b*(*θ*) + 1) ≤ 0, we have *J*(0)*J*(1) < 0, then there is a unique critical point which maximize PDF (2).
  ii- For *k* − *θ*(*b*(*θ*) + 1) > 0 and $\frac{d}{dx}f\left(x\right)<0$, then the PDF (2) is decreasing in *x*.
  **Case II:** *k* > 1
  i- For −4*θ*^2^*b*(*θ*) + *k*^2^ ≥ 0, $k-\sqrt{-4\theta^{2}b(\theta )+k^{2}}>0$ and $\frac{df(x)}{dx}=0$, we have two critical points $x_{0}={\left(\frac{k\pm \sqrt{-4\theta^{2}b(\theta )+k^{2}}}{2\theta }\right)}^{\frac{1}{k-1}}$ which give decreasing–increasing–decreasing PDF (2).
  ii- For −4*θ*^2^*b*(*θ*) + *k*^2^ < 0 and $\frac{d}{dx}f\left(x\right)<0$, then the PDF (2) is decreasing in *x*.

The HRF behavior is described as follows

$$\underset{x\to 0}{\text{lim}}h\left(x\right)=\frac{b\left(\theta \right)\theta^{k+1}}{k!+b\left(\theta \right)\theta^{k}},\;\underset{x\to \infty }{\text{lim}}h\left(x\right)=0.$$

**Proposition 1**. Let *h*(*x*; *θ*, *k*) be the HRF of GTPD, then

I. *h*(*x*: *θ*, *k*) is increasing for *k* ≤ 1.
II. *h*(*x*; *θ*, *k*) is decreasing-increasing for *k* > 1.

**Proof**. According to Glaser lemma [17] and from the PDF (2), we have

$$\rho \left(x\right)=-\frac{f^{{}^{\prime }}\left(x;\theta ,k\right)}{f(x;\theta ,k)}=\frac{\theta b\left(\theta \right)-kx^{k-1}+x^{k}\theta }{b\left(\theta \right)+x^{k}}$$

and

$$\rho^{\prime }\left(x\right)=\frac{kx^{k-2}(b\left(\theta \right)(1-k)+x^{k})}{{\left(b\left(\theta \right)+x^{k}\right)}^{2}},$$

then we conclude that *h*(*x*; *θ*, *k*) is increasing if *k* ≤ 1.

If *k* > 1, *ρ*′(*x*) has a global minimum at some point $x_{0}={\left(b\left(\theta \right)(k-1)\right)}^{\frac{1}{k}}$, then we conclude that *h*(*x*; *θ*, *k*) is decreasing-increasing.

### 3.2 Moments and related measures of GTPD

Let *X* ∼ GTPD, Then the *i*th moment of *X* is determined as follows

$$\begin{array}{c} E\left(X^{i}\right)=\int_{0}^{\infty }x^{i}f\left(x\right)dx=\frac{\theta^{k}b\left(\theta \right)i!+\left(k+i\right)!}{\theta^{k+i}b\left(\theta \right)+\theta^{i}k!}. \end{array}$$ (5)

Hence, the first four moments of the GTPD random variable can be found by substituting *i* = 1, 2, 3, 4, respectively, in Eq (5). They are used to determine variance, Skewness, Kurtosis, and coefficient of variation of GTPD, respectively, as follows.

$$Var\left(X\right)=\frac{\left((k+2)!k!+b^{2}(\theta )\theta^{2k}-(k+1){!}^{2}+2b(\theta )\theta^{k}k!-2b\left(\theta \right)\theta^{k}(k+1)!+b(\theta )\theta^{k}(k+2)!\right)}{\theta^{2}{\left(k!+b(\theta )\theta^{k}\right)}^{2}},$$

$$Skewness=\sqrt{\beta_{1}}=\frac{E(X^{3})}{{\left(Var\left(X\right)\right)}^{\frac{3}{2}}}=\frac{\frac{3!\theta^{k}b\left(\theta \right)+\left(k+3\right)!}{\theta^{k+3}b\left(\theta \right)+\theta^{3}k!}}{{\left(\frac{\left((k+2)!k!+b^{2}(\theta )\theta^{2k}-(k+1){!}^{2}+2b(\theta )\theta^{k}k!-2b\left(\theta \right)\theta^{k}(k+1)!+b(\theta )\theta^{k}(k+2)!\right)}{\theta^{2}{\left(k!+b(\theta )\theta^{k}\right)}^{2}}\right)}^{\frac{3}{2}}},$$

$$Kurtosis=\beta_{2}=\frac{E(X^{4})}{{\left(Var\left(X\right)\right)}^{2}}=\frac{\frac{4!\theta^{k}b\left(\theta \right)+\left(k+4\right)!}{\theta^{k+4}b\left(\theta \right)+\theta^{4}k!}}{{\left(\frac{\left((k+2)!k!+b^{2}(\theta )\theta^{2k}-(k+1){!}^{2}+2b(\theta )\theta^{k}k!-2b\left(\theta \right)\theta^{k}(k+1)!+b(\theta )\theta^{k}(k+2)!\right)}{\theta^{2}{\left(k!+b(\theta )\theta^{k}\right)}^{2}}\right)}^{2}},$$

$$C.V=\gamma =\frac{\sqrt{Var\left(X\right)}}{E\left(X\right)}=\frac{{\left(\frac{\left(\left(k+2\right)!k!+b^{2}\left(\theta \right)\theta^{2k}-\left(k+1\right){!}^{2}+2b\left(\theta \right)\theta^{k}k!-2b\left(\theta \right)\theta^{k}\left(k+1\right)!+b\left(\theta \right)\theta^{k}\left(k+2\right)!\right)}{\theta^{2}{\left(k!+b\left(\theta \right)\theta^{k}\right)}^{2}}\right)}^{\frac{1}{2}}}{\frac{\theta^{k}b\left(\theta \right)+\left(k+1\right)!}{\theta^{k+1}b\left(\theta \right)+\theta k!}}.$$

The moment-generating function of the GTPD is determined as follows

$$M(t)=\int_{0}^{\infty }e^{tx}f(x)dx=\frac{\theta^{k+1}\left(b(\theta )+\Gamma (k+1){\left(\theta -t\right)}^{-k}\right)}{(\theta -t)\left(k!+b(\theta )\theta^{k}\right)},$$ (6)

the GTPD characteristic function *ϕ*(*t*) is obtained by replacing *t* in Eq (6) by *it*.

The *i*th incomplete moments of GTPD are determined as follows

$$T_{i}\left(t\right)=\int_{0}^{t}x^{i}f\left(x\right)dx=\frac{\theta^{-i}\left(b\left(\theta \right)\theta^{k}\left(\Gamma \left(i+1\right)-\Gamma \left(i+1,t\theta \right)\right)-\Gamma \left(i+k+1,t\theta \right)+\Gamma \left(i+k+1\right)\right)}{k!+b\left(\theta \right)\theta^{k}},$$

where $\Gamma \left(a,z\right)=\int_{z}^{\infty }t^{a-1}e^{-t}dt$. We have first incomplete moments *T*_1_(*t*) in the last equation when *i* = 1, which is used to calculate the mean residual life and the mean waiting time which is, respectively, defined as follows

$$\psi \left(t\right)=[1-T_{1}\left(t\right)]/S\left(t\right)-t,$$

$$M_{1}\left(t\right)=t-T_{1}\left(t\right)/F\left(t\right).,$$

Another use of *T*_1_(*t*) is to calculate Bonferroni and Lorenz curves which are, respectively, defined as follows.

$$L\left(p\right)=T_{1}\left(t\right)/E\left(X\right),$$

$$B\left(p\right)=T_{1}\left(x_{p}\right)/\left(pE\left(X\right)\right),$$

Where *x*_*p*_ is the quantile function of GTPD.

### 3.3 Entropy

It is commonly understood that entropy and information can be used to calculate the degree of uncertainty in a probability distribution. However, many correlations have been created based on the features of entropy.

The entropy of a random variable *X* is a measure of the uncertainty’s variation. The entropy of Rényi [18] is defined as follows

$$I_{R}\left(s\right)=\frac{1}{1-s}\text{log}\{\int_{0}^{\infty }f^{s}\left(x\right)dx\},$$

where *s*(*integer*) > 0 and *s* ≠ 1. For the GTPD, we have

$$I_{R}\left(s\right)=\frac{1}{1-s}\text{log}\left(\int_{0}^{\infty }\frac{\theta^{s\left(k+1\right)}}{{\left(\theta^{k}b\left(\theta \right)+k!\right)}^{s}}{\left(b\left(\theta \right)+x^{k}\right)}^{s}e^{-\theta sx}dx\right),$$

by using expansion for (*b*(*θ*) + *x*^*k*^)^*s*^, we have

$$I_{R}\left(s\right)=\frac{1}{1-s}\text{log}\left(\frac{\theta^{s\left(k+1\right)}b\left(\theta \right)}{{\left(\theta^{k}b\left(\theta \right)+k!\right)}^{s}}\sum_{i=0}^{n}\frac{n!}{\left(n-i\right)!i!}b^{i}\left(\theta \right)\frac{\Gamma \left(k\left(n-i\right)+1\right)}{s\theta {\left(s\theta \right)}^{k\left(n-i\right)}}\right),$$

we get Shannon’s entropy as *s* → 1.

### 3.4 Stochastic orders

This section gives some properties of Stochastic order(<_*s*_), Convex order (<_*cx*_), hazard rate order (<_*hr*_) and Likelihood ratio order (<_*lr*_) of two random variables *X*, *Y* from GTPD.

**Proposition 2**. Let *X*_1_ and *X*_2_ ⇝ GTPD (*θ*_*i*_, *k*_*i*_); *i* = 1, 2 be two random variables. If *θ*_1_ ≥ *θ*_2_, *k*_1_ ≤ *k*_2_ and *b*(*θ*_1_) ≥ *b*(*θ*_2_), then *X*_1_ <_*lr*_ *X*_2_, *X*_1_ <_*hr*_ *X*_2_, *X*_1_ <_*s*_ *X*_2_ and *X*_1_ <_*cx*_ *X*_2_.

**Proof**. We have

$$\frac{f_{X_{1}}\left(t\right)}{f_{X_{2}}\left(t\right)}=\frac{h\left(\theta_{1}\right)[b\left(\theta_{1}\right)+t^{k_{1}}]}{h\left(\theta_{2}\right)[b\left(\theta_{2}\right)+t^{k_{2}}]}e^{-\left(\theta_{1}-\theta_{2}\right)t}.$$

For simplification, we use $\text{ln}(\frac{f_{X_{1}}\left(t\right)}{f_{X_{2}}\left(t\right)})$. Now, we can find

$$\text{ln}\left(\frac{f_{X_{1}}\left(t\right)}{f_{X_{2}}\left(t\right)}\right)=\text{ln}\left(h\left(\theta_{1}\right)\right)+\text{ln}[b\left(\theta_{1}\right)+t^{k_{1}}]-\text{ln}\left(h\left(\theta_{2}\right)\right)-\text{ln}[b\left(\theta_{2}\right)+t^{k_{2}}]-\left(\theta_{1}-\theta_{2}\right)t,$$

$$\frac{d}{dt}\text{ln}\left(\frac{f_{X_{1}}\left(t\right)}{f_{X_{2}}\left(t\right)}\right)=\frac{k_{1}t^{k_{1}-1}\left(b\left(\theta_{2}\right)+t^{k_{2}}\right)-k_{2}t^{k_{2}-1}\left(b\left(\theta_{1}\right)+t^{k_{1}}\right)}{\left(b\left(\theta_{1}\right)+t^{k_{1}}\right)\left(b\left(\theta_{2}\right)+t^{k_{2}}\right)}-\left(\theta_{1}-\theta_{2}\right).$$

To this end, If *θ*_1_ ≥ *θ*_2_, *k*_1_ ≤ *k*_2_ and *b*(*θ*_1_) ≥ *b*(*θ*_2_), we have $\frac{d}{dt}\text{ln}\left(\frac{f_{X_{1}}\left(t\right)}{f_{X_{2}}\left(t\right)}\right)\leq 0$. This means that *X*_1_ <_*lr*_ *X*_2_. Also, we have Likelihood ratio order⇒ hazard rate order ⇒ Stochastic order, and *E*[*X*_1_] = *E*[*X*_2_], then convex order ⇔ stochastic order which proved proposition 2.

## 4 Estimation of GTPD parameters

As we will see in this section, there are many different traditional estimation techniques that can be used to acquire an estimate of the GTPD parameters. These parameters can be derived by either maximizing or minimizing an objective function, as we will discuss in detail.

- The parameters of GTPD that have been estimated using the maximum likelihood estimation (MLE) approach are derived by maximizing the log-likelihood function, which has the following definition
  $$\text{ln}\;L\left(x_{i},\theta ,k\right)=n\;\text{ln}\left(h\left(\theta \right)\right)+\sum_{i=1}^{n}\text{ln}\left(b\left(\theta \right)+x_{i}^{k}\right)-\theta \sum_{i=1}^{n}x_{i}.$$
- Using the Anderson-Darling estimation (ADE) approach, the estimated GTPD parameters are obtained by minimizing the following equation (*x*_(1)_ ≤ *x*_(2)_ ≤ … ≤ *x*_(*n*)_)
  $$\begin{array}{cc} A\left(x_{i},\theta ,k\right) & =-n-\frac{1}{n}\sum_{i=1}^{n}\left(2i-1\right)\left[\text{log}\;F\left(x_{i}\right)+\text{log}\;S\left(x_{i}\right)\right]\\ & =-n-\frac{1}{n}\sum_{i=1}^{n}\left(2i-1\right)[\text{log}\frac{\theta^{k}[b\left(\theta \right)e^{-\theta x_{i}}+\theta \Gamma \left(k+1,x_{i}\theta \right)]}{\theta^{k}b\left(\theta \right)+k!}+\text{log}\left(1-\frac{\theta^{k}[b\left(\theta \right)e^{-\theta x_{i}}+\theta \Gamma \left(k+1,x_{i}\theta \right)]}{\theta^{k}b\left(\theta \right)+k!}\right)]. \end{array}$$
- Using the right-tail Anderson-Darling estimation (RADE) approach, the estimated GTPD parameters are obtained by minimizing the following equation (*x*_(1)_ ≤ *x*_(2)_ ≤ … ≤ *x*_(*n*)_)
  $$\begin{array}{cc} R\left(x_{i},\theta ,k\right) & =\frac{n}{2}-2\sum_{i=1}^{n}F\left(x_{i}\right)-\frac{1}{n}\sum_{i=1}^{n}\left(2i-1\right)\text{log}\;S\left(x_{n+1-i}\right)=\frac{n}{2}-2\sum_{i=1}^{n}\frac{\theta^{k}[b\left(\theta \right)e^{-\theta x_{i}}+\theta \Gamma \left(k+1,x_{i}\theta \right)]}{\theta^{k}b\left(\theta \right)+k!}\\ & -\frac{1}{n}\sum_{i=1}^{n}\left(2i-1\right)\text{log}\left(1-\frac{\theta^{k}[b\left(\theta \right)e^{-\theta x_{n+1-i}}+\theta \Gamma \left(k+1,x_{n+1-i}\theta \right)]}{\theta^{k}b\left(\theta \right)+k!}\right). \end{array}$$
- Using the left-tailed Anderson-Darling estimation (LTADE) approach, the estimated GTPD parameters are obtained by minimizing the following equation (*x*_(1)_ ≤ *x*_(2)_ ≤ … ≤ *x*_(*n*)_)
  $$\begin{array}{cc} L\left(x_{i},\theta ,k\right) & =-\frac{3}{2}n+2\sum_{i=1}^{n}F\left(x_{i}\right)-\frac{1}{n}\sum_{i=1}^{n}\left(2i-1\right)\text{log}\;F\left(x_{i}\right)\\ & =-\frac{3}{2}n+2\sum_{i=1}^{n}\frac{\theta^{k}[b\left(\theta \right)e^{-\theta x_{i}}+\theta \Gamma \left(k+1,x_{i}\theta \right)]}{\theta^{k}b\left(\theta \right)+k!}-\frac{1}{n}\sum_{i=1}^{n}\left(2i-1\right)\text{log}\frac{\theta^{k}[b\left(\theta \right)e^{-\theta x_{i}}+\theta \Gamma \left(k+1,x_{i}\theta \right)]}{\theta^{k}b\left(\theta \right)+k!}. \end{array}$$
- Using the Cramér-von Mises estimation (CVME) approach, the estimated GTPD parameters are obtained by minimizing the following equation (*x*_(1)_ ≤ *x*_(2)_ ≤ … ≤ *x*_(*n*)_)
  $$C\left(x_{i},\theta ,k\right)=-\frac{1}{12n}+\sum_{i=1}^{n}{\left[F\left(x_{i}\right)-\frac{2i-1}{2n}\right]}^{2}=-\frac{1}{12n}+\sum_{i=1}^{n}{\left[\frac{\theta^{k}[b\left(\theta \right)e^{-\theta x_{i}}+\theta \Gamma \left(k+1,x_{i}\theta \right)]}{\theta^{k}b\left(\theta \right)+k!}-\frac{2i-1}{2n}\right]}^{2}.$$
- Using the least-squares estimation (LSE) approach, the estimated GTPD parameters are obtained by minimizing the following equation (*x*_(1)_ ≤ *x*_(2)_ ≤ … ≤ *x*_(*n*)_)
  $$V\left(x_{i},\theta ,k\right)=\sum_{i=1}^{n}{\left[F\left(x_{i}\right)-\frac{i}{n+1}\right]}^{2}=\sum_{i=1}^{n}{\left[\frac{\theta^{k}[b\left(\theta \right)e^{-\theta x_{i}}+\theta \Gamma \left(k+1,x_{i}\theta \right)]}{\theta^{k}b\left(\theta \right)+k!}-\frac{i}{n+1}\right]}^{2}.$$
- Using the weighted least-squares estimation (WLSE) approach, the estimated GTPD parameters are obtained by minimizing the following equation (*x*_(1)_ ≤ *x*_(2)_ ≤ … ≤ *x*_(*n*)_)
  $$\begin{array}{cc} W\left(x_{i},\theta ,k\right) & =\sum_{i=1}^{n}\frac{{\left(n+1\right)}^{2}\left(n+2\right)}{i\left(n-i+1\right)}{\left[F\left(x_{i}\right)-\frac{i}{n+1}\right]}^{2}\\ & =\sum_{i=1}^{n}\frac{{\left(n+1\right)}^{2}\left(n+2\right)}{i\left(n-i+1\right)}{\left[\frac{\theta^{k}[b\left(\theta \right)e^{-\theta x_{i}}+\theta \Gamma \left(k+1,x_{i}\theta \right)]}{\theta^{k}b\left(\theta \right)+k!}-\frac{i}{n+1}\right]}^{2}. \end{array}$$
- Using the maximum product of spacing estimation (MPSE) approach, the estimated GTPD parameters are obtained by maximizing the following equation (*x*_(1)_ ≤ *x*_(2)_ ≤ … ≤ *x*_(*n*)_)
  $$H\left(x_{i},\theta ,k\right)=\frac{1}{n+1}\sum_{i=1}^{n+1}\text{log}\;D_{i}\left(x_{i},\theta ,k\right),$$
  where
  $$\begin{array}{cc} D_{i}\left(\alpha ,\lambda ,\xi \right) & =F\left(x_{i}\right)-F\left(x_{i-1}\right)\\ & =\frac{\theta^{k}}{\theta^{k}b\left(\theta \right)+k!}\{[b\left(\theta \right)e^{-\theta x_{i}}+\theta \Gamma \left(k+1,x_{i}\theta \right)]-[b\left(\theta \right)e^{-\theta x_{i-1}}+\theta \Gamma \left(k+1,x_{i-1}\theta \right)]\}. \end{array}$$
- Using the minimum spacing absolute distance estimation (MSADE) approach, the estimated GTPD parameters are obtained by minimizing the following equation (*x*_(1)_ ≤ *x*_(2)_ ≤ … ≤ *x*_(*n*)_)
  $$M_{1}\left(x_{i},\theta ,k\right)=\sum_{i=1}^{n+1}\left|D_{i}-\frac{1}{n+1}\right|.$$
- Using the minimum spacing absolute-log distance estimation method (MSALDE) approach, the estimated GTPD parameters are obtained by minimizing the following equation (*x*_(1)_ ≤ *x*_(2)_ ≤ … ≤ *x*_(*n*)_)
  $$M_{2}\left(x_{i},\theta ,k\right)=\sum_{i=1}^{n+1}\left|\text{log}\;D_{i}-\text{log}\frac{1}{n+1}\right|.$$

## 5 Special case of GTPD

In this section, we presented a new statistical distribution called Gemeay distribution (GD) which is a special case of GTPD, and it is obtained by taking *b*(*θ*) = *θ*^−2^. The PDF, CDF, and HRF of GD are, respectively, defined as follows

$$\begin{array}{c} f\left(x\right)=\frac{\theta^{k+1}e^{-\theta x}\left(\frac{1}{\theta^{2}}+x^{k}\right)}{k!+\theta^{k-2}}, \end{array}$$ (7)

$$\begin{array}{c} F\left(x\right)=\frac{\theta^{k}\left(1-e^{\theta \left(-x\right)}\right)+\theta^{2}\Gamma \left(k+1,x\theta \right)}{\theta^{2}k!+\theta^{k}}, \end{array}$$ (8)

$$\begin{array}{c} h\left(x\right)=\frac{\theta +\theta^{3}x^{k}}{\theta^{2-k}e^{\theta x}\Gamma \left(k+1,x\theta \right)+1}. \end{array}$$ (9)

**Fig 1:**
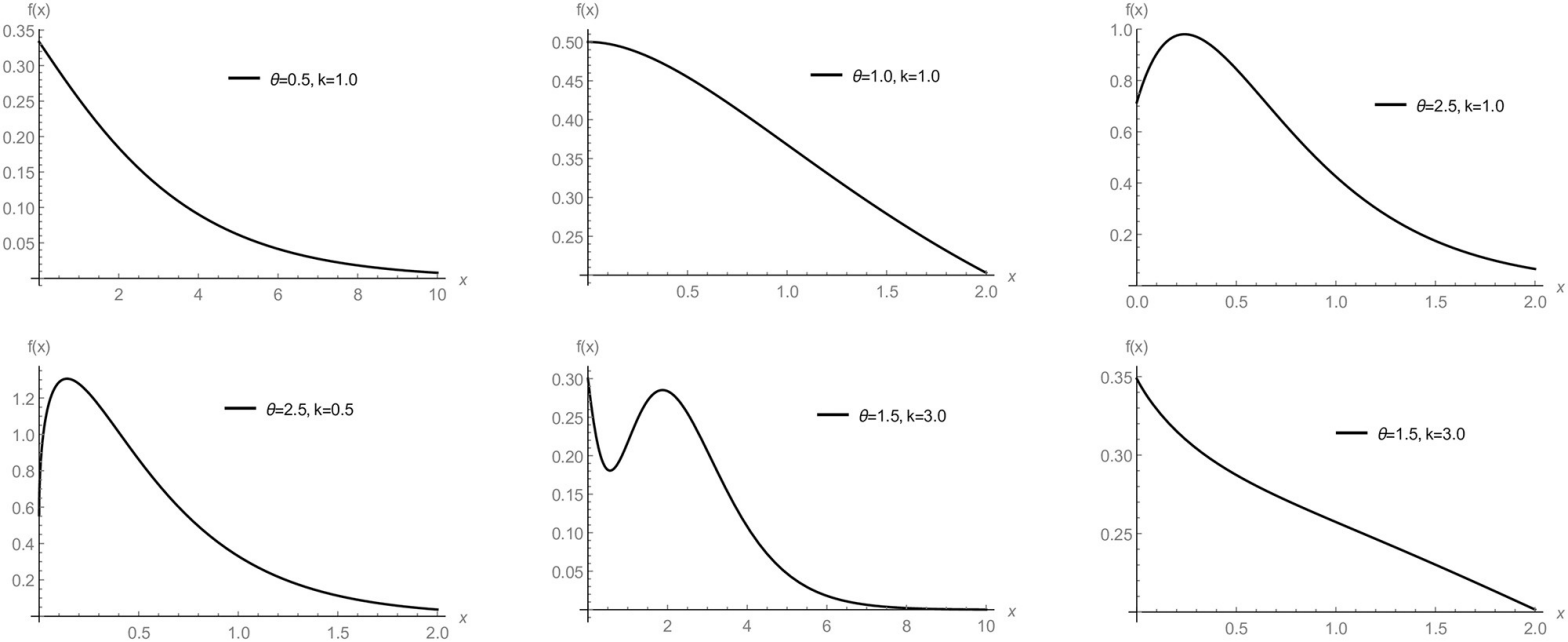
Plots of the PDF of the GD with various parametric values.

**Fig 2:**
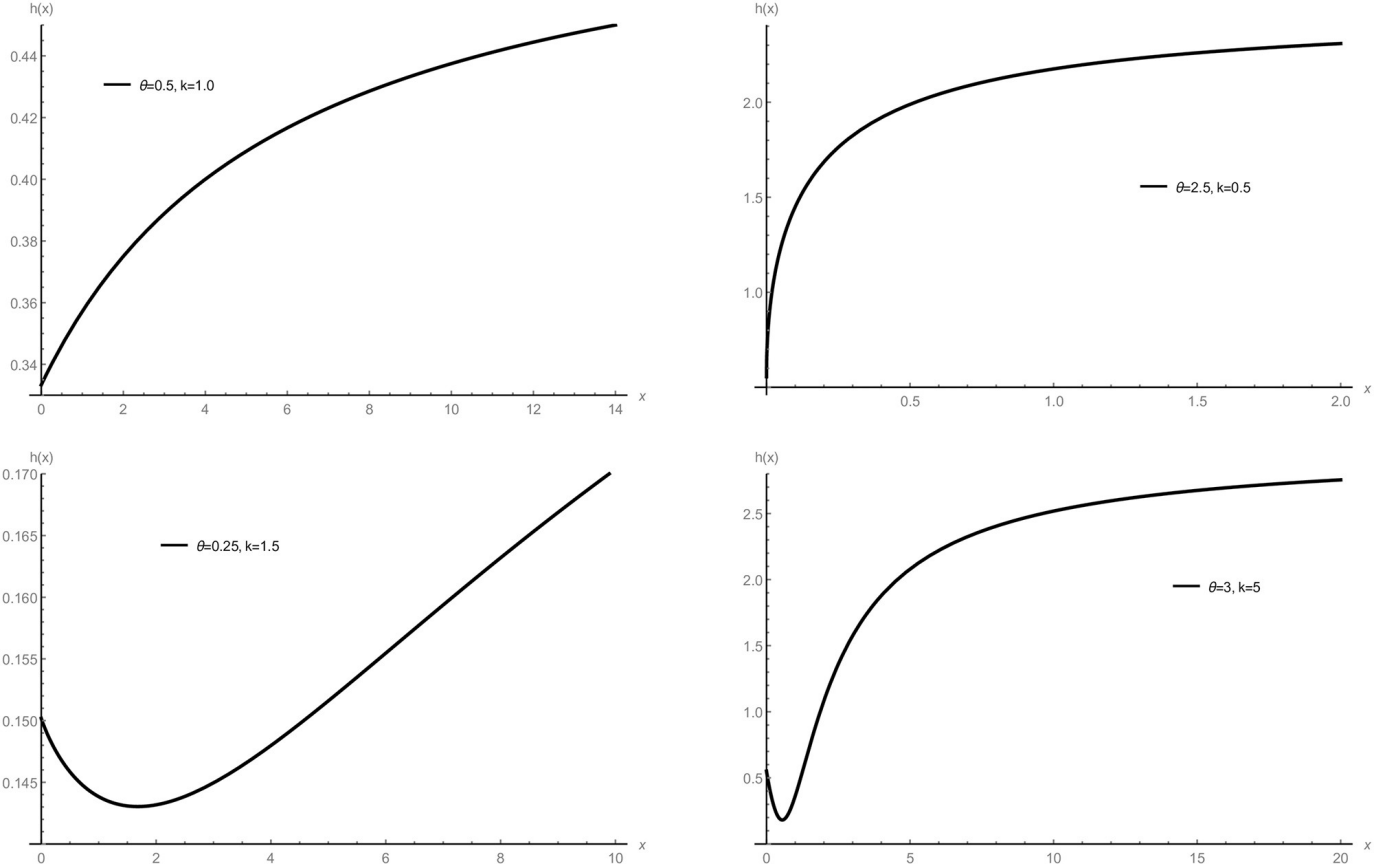
Plots of the HRF of the GD with various parametric values.

Plots of PDF 7 are presented in Fig 1, which deal with the study of the behavior of the GTPD in Subsection 3.1 when replacing *b*(*θ*) by $\frac{1}{\theta^{2}}$. The HRF 9 of GD are presented graphically in Fig 2, which deal with results proofed in Proposition 1 when replacing *b*(*θ*) by $\frac{1}{\theta^{2}}$.

## 6 Numerical simulation

In this section, we will use all of the estimating techniques discussed in Section 4, but we will substitute $\frac{1}{\theta^{2}}$ for *b*(*θ*). Now that we have these different estimating techniques, we will investigate how well they function when used to estimate the parameters of the GD. In addition to this, we evaluate a comparison of each approach by comparing the numerical values of the average of Bias (BIAS) $|Bias\left(\hat{\Omega }\right)|=\frac{1}{M}\sum_{i=1}^{M}\left|\hat{\Omega }-\Omega \right|$, mean squared errors (MSE), $MSE=\frac{1}{M}\sum_{i=1}^{M}{\left(\hat{\Omega }-\Omega \right)}^{2}$, and mean relative errors (MRE) $MRE=\frac{1}{M}\sum_{i=1}^{M}\left|\hat{\Omega }-\Omega \right|/\Omega$, ***Ω*** = (*θ*, *k*). Simulation results may be used to choose the optimal estimation method for model parameters. The R software is used to have *M* = 10000 random samples from GD for sample sizes equal to 30, 75, 150, 250, 400, and 600.

The numerical results of the simulations are shown in Tables 1–5, and the power of each number relates to its order when compared to the other estimating techniques along the same line. Table 6 shows our estimators’ partial and total rankings. We find that MPSE is the best approach for estimating suggested model parameters when using random samples from our model.

**Table 1:** Simulation values of BIAS, MSE and MRE for (*θ* = 0.25, *k* = 0.5).

n	Est.	Est. Par.	MLE	ADE	CVME	MPSE	LSE	RTADE	WLSE	LTADE	MSADE	MSALDE
30	BIAS	$\hat{\theta }$	0.364336^{7}^	0.300791^{4}^	0.471379^{9}^	0.247877^{2}^	0.371279^{8}^	0.339218^{5}^	0.342416^{6}^	0.478417^{10}^	0.254735^{3}^	0.239141^{1}^
		$\hat{k}$	0.693861^{7}^	0.637158^{4}^	0.887113^{10}^	0.48413^{3}^	0.709915^{9}^	0.703311^{8}^	0.675725^{6}^	0.655392^{5}^	0.455141^{1}^	0.477669^{2}^
	MSE	$\hat{\theta }$	0.374379^{7}^	0.22781^{4}^	0.591415^{10}^	0.208485^{3}^	0.475077^{9}^	0.331445^{5}^	0.354996^{6}^	0.442738^{8}^	0.190575^{2}^	0.143221^{1}^
		$\hat{k}$	1.120406^{6}^	0.885173^{5}^	1.823321^{10}^	0.67336^{3}^	1.480426^{9}^	1.178243^{8}^	1.14833^{7}^	0.780955^{4}^	0.64706^{2}^	0.589474^{1}^
	MRE	$\hat{\theta }$	0.485781^{7}^	0.401054^{4}^	0.628505^{9}^	0.330503^{2}^	0.495039^{8}^	0.452291^{5}^	0.456555^{6}^	0.637889^{10}^	0.339646^{3}^	0.318854^{1}^
		$\hat{k}$	2.775446^{7}^	2.548634^{4}^	3.548451^{10}^	1.936519^{3}^	2.83966^{9}^	2.813246^{8}^	2.7029^{6}^	2.621569^{5}^	1.820563^{1}^	1.910677^{2}^
	∑ *Ranks*		41^{7}^	25^{4}^	58^{10}^	16^{3}^	52^{9}^	39^{6}^	37^{5}^	42^{8}^	12^{2}^	8^{1}^
75	BIAS	$\hat{\theta }$	0.192213^{6}^	0.207538^{7}^	0.25977^{9}^	0.135797^{1}^	0.237914^{8}^	0.188737^{5}^	0.187403^{4}^	0.274221^{10}^	0.181857^{3}^	0.155649^{2}^
		$\hat{k}$	0.441739^{5}^	0.477487^{7}^	0.548115^{10}^	0.334986^{1}^	0.517873^{9}^	0.463408^{6}^	0.432202^{4}^	0.487955^{8}^	0.375514^{3}^	0.366503^{2}^
	MSE	$\hat{\theta }$	0.083931^{4}^	0.106106^{7}^	0.173518^{9}^	0.045055^{1}^	0.141378^{8}^	0.082059^{3}^	0.085791^{5}^	0.17743^{10}^	0.085848^{6}^	0.056398^{2}^
		$\hat{k}$	0.376884^{5}^	0.428866^{7}^	0.615598^{10}^	0.216546^{1}^	0.515049^{9}^	0.397351^{6}^	0.366357^{4}^	0.437517^{8}^	0.359134^{3}^	0.277218^{2}^
	MRE	$\hat{\theta }$	0.256285^{6}^	0.276717^{7}^	0.346361^{9}^	0.181063^{1}^	0.317218^{8}^	0.251649^{5}^	0.249871^{4}^	0.365628^{10}^	0.242476^{3}^	0.207532^{2}^
		$\hat{k}$	1.766956^{5}^	1.909947^{7}^	2.192461^{10}^	1.339943^{1}^	2.071493^{9}^	1.853631^{6}^	1.728806^{4}^	1.95182^{8}^	1.502057^{3}^	1.466013^{2}^
	∑ *Ranks*		31^{5.5}^	42^{7}^	57^{10}^	6^{1}^	51^{8}^	31^{5.5}^	25^{4}^	54^{9}^	21^{3}^	12^{2}^
150	BIAS	$\hat{\theta }$	0.126236^{4}^	0.130092^{5}^	0.160128^{9}^	0.104627^{1}^	0.13736^{8}^	0.134768^{7}^	0.130445^{6}^	0.169728^{10}^	0.126113^{3}^	0.122401^{2}^
		$\hat{k}$	0.331631^{4}^	0.345244^{8}^	0.386833^{10}^	0.289109^{1}^	0.344275^{6}^	0.36122^{9}^	0.341205^{5}^	0.344692^{7}^	0.297456^{2}^	0.321978^{3}^
	MSE	$\hat{\theta }$	0.035297^{3}^	0.039506^{7}^	0.059478^{9}^	0.022969^{1}^	0.045145^{8}^	0.038922^{5}^	0.038353^{4}^	0.062191^{10}^	0.038925^{6}^	0.032973^{2}^
		$\hat{k}$	0.199482^{4}^	0.211803^{7}^	0.275914^{10}^	0.150281^{1}^	0.211893^{8}^	0.227844^{9}^	0.202879^{6}^	0.200276^{5}^	0.183683^{2}^	0.193626^{3}^
	MRE	$\hat{\theta }$	0.168314^{4}^	0.173456^{5}^	0.213504^{9}^	0.139503^{1}^	0.183147^{8}^	0.179691^{7}^	0.173927^{6}^	0.226304^{10}^	0.16815^{3}^	0.163202^{2}^
		$\hat{k}$	1.326524^{4}^	1.380974^{8}^	1.547332^{10}^	1.156436^{1}^	1.377098^{6}^	1.444879^{9}^	1.364818^{5}^	1.378766^{7}^	1.189822^{2}^	1.287912^{3}^
	∑ *Ranks*		23^{4}^	40^{6}^	57^{10}^	6^{1}^	44^{7}^	46^{8}^	32^{5}^	49^{9}^	18^{3}^	15^{2}^
250	BIAS	$\hat{\theta }$	0.094287^{2}^	0.103932^{5}^	0.127738^{10}^	0.079164^{1}^	0.120192^{8}^	0.100347^{3}^	0.109632^{7}^	0.121066^{9}^	0.108176^{6}^	0.10136^{4}^
		$\hat{k}$	0.271786^{2}^	0.283859^{5}^	0.333529^{10}^	0.228094^{1}^	0.31182^{9}^	0.283027^{4}^	0.294928^{7}^	0.295234^{8}^	0.293576^{6}^	0.281268^{3}^
	MSE	$\hat{\theta }$	0.019278^{2}^	0.023873^{5}^	0.03789^{10}^	0.011705^{1}^	0.030837^{8}^	0.019463^{3}^	0.025468^{6}^	0.034274^{9}^	0.026166^{7}^	0.021488^{4}^
		$\hat{k}$	0.128904^{2}^	0.14135^{6}^	0.196378^{10}^	0.087767^{1}^	0.165629^{9}^	0.131519^{3}^	0.149848^{7}^	0.141048^{5}^	0.162672^{8}^	0.138323^{4}^
	MRE	$\hat{\theta }$	0.125716^{2}^	0.138575^{5}^	0.170318^{10}^	0.105551^{1}^	0.160256^{8}^	0.133795^{3}^	0.146176^{7}^	0.161421^{9}^	0.144235^{6}^	0.135147^{4}^
		$\hat{k}$	1.087142^{2}^	1.135435^{5}^	1.334115^{10}^	0.912375^{1}^	1.247282^{9}^	1.132108^{4}^	1.179711^{7}^	1.180937^{8}^	1.174304^{6}^	1.125072^{3}^
	∑ *Ranks*		12^{2}^	31^{5}^	60^{10}^	6^{1}^	51^{9}^	20^{3}^	41^{7}^	48^{8}^	39^{6}^	22^{4}^
400	BIAS	$\hat{\theta }$	0.076899^{5}^	0.078981^{6}^	0.095053^{9}^	0.063138^{1}^	0.083051^{7}^	0.07617^{4}^	0.075303^{2}^	0.097205^{10}^	0.087962^{8}^	0.075621^{3}^
		$\hat{k}$	0.233784^{8}^	0.231746^{6}^	0.267481^{10}^	0.181406^{1}^	0.231053^{5}^	0.23098^{4}^	0.2178^{2}^	0.24905^{9}^	0.232002^{7}^	0.230603^{3}^
	MSE	$\hat{\theta }$	0.011133^{4}^	0.012198^{5}^	0.017687^{9}^	0.007028^{1}^	0.01375^{7}^	0.011054^{2}^	0.012327^{6}^	0.020246^{10}^	0.015897^{8}^	0.0111^{3}^
		$\hat{k}$	0.089353^{6}^	0.087179^{5}^	0.112723^{10}^	0.061602^{1}^	0.090405^{7}^	0.0865^{3}^	0.078945^{2}^	0.099887^{8}^	0.102051^{9}^	0.086683^{4}^
	MRE	$\hat{\theta }$	0.102532^{5}^	0.105308^{6}^	0.126737^{9}^	0.084184^{1}^	0.110735^{7}^	0.101559^{4}^	0.100403^{2}^	0.129607^{10}^	0.117283^{8}^	0.100828^{3}^
		$\hat{k}$	0.935137^{8}^	0.926985^{6}^	1.069925^{10}^	0.725623^{1}^	0.92421^{5}^	0.923921^{4}^	0.8712^{2}^	0.996201^{9}^	0.928009^{7}^	0.922414^{3}^
	∑ *Ranks*		36^{6}^	34^{5}^	57^{10}^	6^{1}^	38^{7}^	21^{4}^	16^{2}^	56^{9}^	47^{8}^	19^{3}^
600	BIAS	$\hat{\theta }$	0.063585^{3}^	0.065231^{5}^	0.073209^{8}^	0.055511^{1}^	0.077373^{9}^	0.064685^{4}^	0.062148^{2}^	0.082397^{10}^	0.068638^{7}^	0.066215^{6}^
		$\hat{k}$	0.193175^{3}^	0.198741^{4}^	0.213457^{8}^	0.157782^{1}^	0.220199^{10}^	0.203086^{7}^	0.182137^{2}^	0.215801^{9}^	0.202363^{6}^	0.201926^{5}^
	MSE	$\hat{\theta }$	0.007541^{3}^	0.007735^{4}^	0.009979^{8}^	0.005155^{1}^	0.01205^{9}^	0.007991^{6}^	0.007247^{2}^	0.013366^{10}^	0.009932^{7}^	0.007851^{5}^
		$\hat{k}$	0.059951^{3}^	0.062452^{4}^	0.071827^{7}^	0.047085^{1}^	0.081646^{10}^	0.06621^{6}^	0.055058^{2}^	0.075028^{9}^	0.07378^{8}^	0.064616^{5}^
	MRE	$\hat{\theta }$	0.084781^{3}^	0.086975^{5}^	0.097611^{8}^	0.074014^{1}^	0.103164^{9}^	0.086247^{4}^	0.082864^{2}^	0.109862^{10}^	0.091517^{7}^	0.088286^{6}^
		$\hat{k}$	0.772701^{3}^	0.794965^{4}^	0.853829^{8}^	0.631129^{1}^	0.880796^{10}^	0.812345^{7}^	0.728549^{2}^	0.863205^{9}^	0.809454^{6}^	0.807704^{5}^
	∑ *Ranks*		18^{3}^	26^{4}^	47^{8}^	6^{1}^	57^{9.5}^	34^{6}^	12^{2}^	57^{9.5}^	41^{7}^	32^{5}^

**Table 2:** Simulation values of BIAS, MSE and MRE for (*θ* = 0.5, *k* = 1.5).

n	Est.	Est. Par.	MLE	ADE	CVME	MPSE	LSE	RTADE	WLSE	LTADE	MSADE	MSALDE
30	BIAS	$\hat{\theta }$	0.159325^{6}^	0.158221^{5}^	0.188457^{9}^	0.157553^{3}^	0.180033^{8}^	0.157918^{4}^	0.171176^{7}^	0.206033^{10}^	0.152519^{1}^	0.154677^{2}^
		$\hat{k}$	0.577893^{2}^	0.666151^{6}^	0.71829^{7}^	0.753067^{8}^	0.837124^{10}^	0.653345^{5}^	0.759569^{9}^	0.647187^{4}^	0.46602^{1}^	0.617611^{3}^
	MSE	$\hat{\theta }$	0.044759^{6}^	0.04094^{4}^	0.063063^{9}^	0.035862^{1}^	0.049148^{8}^	0.039065^{3}^	0.046978^{7}^	0.083194^{10}^	0.042276^{5}^	0.036339^{2}^
		$\hat{k}$	0.559049^{2}^	0.719775^{5}^	0.82463^{7}^	0.877479^{8}^	1.0256^{10}^	0.702234^{4}^	0.890154^{9}^	0.750628^{6}^	0.417943^{1}^	0.624291^{3}^
	MRE	$\hat{\theta }$	0.31865^{6}^	0.316443^{5}^	0.376914^{9}^	0.315105^{3}^	0.360066^{8}^	0.315836^{4}^	0.342353^{7}^	0.412067^{10}^	0.305038^{1}^	0.309354^{2}^
		$\hat{k}$	0.385262^{2}^	0.444101^{6}^	0.47886^{7}^	0.502044^{8}^	0.558083^{10}^	0.435564^{5}^	0.506379^{9}^	0.431458^{4}^	0.31068^{1}^	0.411741^{3}^
	∑ *Ranks*		24^{3}^	31^{5.5}^	48^{8.5}^	31^{5.5}^	54^{10}^	25^{4}^	48^{8.5}^	44^{7}^	10^{1}^	15^{2}^
75	BIAS	$\hat{\theta }$	0.107738^{1}^	0.117017^{5}^	0.134233^{9}^	0.116231^{4}^	0.128671^{8}^	0.11291^{2}^	0.12258^{7}^	0.140066^{10}^	0.114573^{3}^	0.119037^{6}^
		$\hat{k}$	0.397111^{1}^	0.483797^{5}^	0.546831^{9}^	0.516907^{7}^	0.570615^{10}^	0.456096^{3}^	0.517222^{8}^	0.467758^{4}^	0.417949^{2}^	0.486368^{6}^
	MSE	$\hat{\theta }$	0.018232^{1}^	0.020592^{4}^	0.028362^{9}^	0.019585^{3}^	0.023852^{8}^	0.019291^{2}^	0.022298^{7}^	0.032315^{10}^	0.021422^{6}^	0.020881^{5}^
		$\hat{k}$	0.294749^{1}^	0.463027^{6}^	0.552581^{9}^	0.49752^{7}^	0.599334^{10}^	0.417064^{3}^	0.503206^{8}^	0.456207^{5}^	0.347397^{2}^	0.434946^{4}^
	MRE	$\hat{\theta }$	0.215477^{1}^	0.234034^{5}^	0.268466^{9}^	0.232461^{4}^	0.257343^{8}^	0.225819^{2}^	0.24516^{7}^	0.280132^{10}^	0.229145^{3}^	0.238073^{6}^
		$\hat{k}$	0.26474^{1}^	0.322532^{5}^	0.364554^{9}^	0.344605^{7}^	0.38041^{10}^	0.304064^{3}^	0.344815^{8}^	0.311839^{4}^	0.278633^{2}^	0.324245^{6}^
	∑ *Ranks*		6^{1}^	30^{4}^	54^{9.5}^	32^{5}^	54^{9.5}^	15^{2}^	45^{8}^	43^{7}^	18^{3}^	33^{6}^
150	BIAS	$\hat{\theta }$	0.077767^{1}^	0.086625^{4}^	0.101612^{10}^	0.083377^{3}^	0.098634^{8}^	0.080272^{2}^	0.090111^{5}^	0.101159^{9}^	0.090655^{7}^	0.090591^{6}^
		$\hat{k}$	0.271437^{1}^	0.346258^{6}^	0.387038^{9}^	0.337787^{4}^	0.409674^{10}^	0.319798^{2}^	0.357978^{7}^	0.34291^{5}^	0.322365^{3}^	0.362237^{8}^
	MSE	$\hat{\theta }$	0.009767^{1}^	0.011783^{4}^	0.016132^{9}^	0.010979^{3}^	0.01518^{8}^	0.010488^{2}^	0.012597^{6}^	0.016465^{10}^	0.01314^{7}^	0.012464^{5}^
		$\hat{k}$	0.15874^{1}^	0.277196^{5}^	0.330589^{9}^	0.261414^{4}^	0.379445^{10}^	0.236287^{3}^	0.287982^{8}^	0.278693^{7}^	0.232933^{2}^	0.277308^{6}^
	MRE	$\hat{\theta }$	0.155535^{1}^	0.17325^{4}^	0.203223^{10}^	0.166755^{3}^	0.197269^{8}^	0.160545^{2}^	0.180223^{5}^	0.202318^{9}^	0.18131^{7}^	0.181182^{6}^
		$\hat{k}$	0.180958^{1}^	0.230838^{6}^	0.258026^{9}^	0.225192^{4}^	0.273116^{10}^	0.213198^{2}^	0.238652^{7}^	0.228606^{5}^	0.21491^{3}^	0.241491^{8}^
	∑ *Ranks*		6^{1}^	29^{4.5}^	56^{10}^	21^{3}^	54^{9}^	13^{2}^	38^{6}^	45^{8}^	29^{4.5}^	39^{7}^
250	BIAS	$\hat{\theta }$	0.057482^{1}^	0.066939^{4}^	0.079546^{9}^	0.065819^{3}^	0.077937^{8}^	0.062765^{2}^	0.067735^{5}^	0.08678^{10}^	0.073733^{7}^	0.070487^{6}^
		$\hat{k}$	0.192552^{1}^	0.243482^{3}^	0.287444^{8}^	0.246683^{4}^	0.296126^{10}^	0.219624^{2}^	0.253235^{5}^	0.290706^{9}^	0.263876^{7}^	0.257966^{6}^
	MSE	$\hat{\theta }$	0.005384^{1}^	0.007255^{4}^	0.010102^{9}^	0.006931^{3}^	0.009883^{8}^	0.006482^{2}^	0.007405^{5}^	0.011586^{10}^	0.008946^{7}^	0.008059^{6}^
		$\hat{k}$	0.074191^{1}^	0.142381^{4}^	0.208142^{9}^	0.136451^{3}^	0.226483^{10}^	0.11686^{2}^	0.156808^{7}^	0.204951^{8}^	0.154833^{6}^	0.142698^{5}^
	MRE	$\hat{\theta }$	0.114964^{1}^	0.133877^{4}^	0.159092^{9}^	0.131638^{3}^	0.155873^{8}^	0.12553^{2}^	0.135469^{5}^	0.17356^{10}^	0.147466^{7}^	0.140974^{6}^
		$\hat{k}$	0.128368^{1}^	0.162321^{3}^	0.191629^{8}^	0.164455^{4}^	0.197418^{10}^	0.146416^{2}^	0.168823^{5}^	0.193804^{9}^	0.175917^{7}^	0.171977^{6}^
	∑ *Ranks*		6^{1}^	22^{4}^	52^{8}^	20^{3}^	54^{9}^	12^{2}^	32^{5}^	56^{10}^	41^{7}^	35^{6}^
400	BIAS	$\hat{\theta }$	0.047175^{1}^	0.052576^{4}^	0.063403^{8}^	0.048835^{3}^	0.065022^{9}^	0.047873^{2}^	0.053841^{5}^	0.068039^{10}^	0.061008^{7}^	0.054212^{6}^
		$\hat{k}$	0.149347^{1}^	0.179933^{5}^	0.223455^{8}^	0.16232^{3}^	0.23748^{10}^	0.157135^{2}^	0.179374^{4}^	0.224233^{9}^	0.203791^{7}^	0.191368^{6}^
	MSE	$\hat{\theta }$	0.003614^{1}^	0.004572^{4}^	0.006639^{8}^	0.003885^{3}^	0.00717^{9}^	0.003774^{2}^	0.004794^{5}^	0.007442^{10}^	0.00603^{7}^	0.004886^{6}^
		$\hat{k}$	0.042856^{1}^	0.071322^{4}^	0.131055^{8}^	0.055576^{3}^	0.153089^{10}^	0.052666^{2}^	0.077461^{5}^	0.133332^{9}^	0.089026^{7}^	0.078676^{6}^
	MRE	$\hat{\theta }$	0.094351^{1}^	0.105153^{4}^	0.126807^{8}^	0.097671^{3}^	0.130044^{9}^	0.095746^{2}^	0.107683^{5}^	0.136077^{10}^	0.122017^{7}^	0.108423^{6}^
		$\hat{k}$	0.099565^{1}^	0.119955^{5}^	0.14897^{8}^	0.108213^{3}^	0.15832^{10}^	0.104757^{2}^	0.119583^{4}^	0.149489^{9}^	0.13586^{7}^	0.127579^{6}^
	∑ *Ranks*		6^{1}^	26^{4}^	48^{8}^	18^{3}^	57^{9.5}^	12^{2}^	28^{5}^	57^{9.5}^	42^{7}^	36^{6}^
600	BIAS	$\hat{\theta }$	0.037929^{1}^	0.04218^{4}^	0.053255^{9}^	0.039353^{2}^	0.052632^{8}^	0.040077^{3}^	0.04386^{5}^	0.055103^{10}^	0.047349^{7}^	0.045026^{6}^
		$\hat{k}$	0.116223^{1}^	0.135648^{4}^	0.1754^{10}^	0.121266^{2}^	0.172672^{9}^	0.128422^{3}^	0.145493^{5}^	0.168226^{8}^	0.145936^{6}^	0.151216^{7}^
	MSE	$\hat{\theta }$	0.002269^{1}^	0.002979^{4}^	0.004803^{10}^	0.002508^{2}^	0.00452^{8}^	0.002555^{3}^	0.003145^{5}^	0.0048^{9}^	0.003644^{7}^	0.003371^{6}^
		$\hat{k}$	0.023194^{1}^	0.038208^{4}^	0.083421^{10}^	0.029328^{3}^	0.074726^{9}^	0.027973^{2}^	0.044813^{6}^	0.068815^{8}^	0.03867^{5}^	0.04693^{7}^
	MRE	$\hat{\theta }$	0.075857^{1}^	0.084359^{4}^	0.10651^{9}^	0.078705^{2}^	0.105265^{8}^	0.080155^{3}^	0.087719^{5}^	0.110206^{10}^	0.094698^{7}^	0.090053^{6}^
		$\hat{k}$	0.077482^{1}^	0.090432^{4}^	0.116933^{10}^	0.080844^{2}^	0.115114^{9}^	0.085614^{3}^	0.096995^{5}^	0.112151^{8}^	0.097291^{6}^	0.100811^{7}^
	∑ *Ranks*		6^{1}^	24^{4}^	58^{10}^	13^{2}^	51^{8}^	17^{3}^	31^{5}^	53^{9}^	38^{6}^	39^{7}^

**Table 3:** Simulation values of BIAS, MSE and MRE for (*θ* = 2.5, *k* = 0.5).

n	Est.	Est. Par.	MLE	ADE	CVME	MPSE	LSE	RTADE	WLSE	LTADE	MSADE	MSALDE
30	BIAS	$\hat{\theta }$	0.785294^{8}^	0.702178^{2}^	0.767514^{7}^	0.75047^{5}^	0.766235^{6}^	0.802266^{10}^	0.700936^{1}^	0.793833^{9}^	0.711288^{3}^	0.726527^{4}^
		$\hat{k}$	0.574641^{9}^	0.512133^{4}^	0.548032^{7}^	0.557473^{8}^	0.535398^{6}^	0.585492^{10}^	0.508747^{3}^	0.489018^{1}^	0.503761^{2}^	0.531362^{5}^
	MSE	$\hat{\theta }$	1.071863^{10}^	0.782123^{1}^	0.931719^{8}^	0.899174^{5}^	0.907016^{7}^	1.042924^{9}^	0.822441^{2}^	0.90266^{6}^	0.876687^{4}^	0.830648^{3}^
		$\hat{k}$	0.632446^{10}^	0.44092^{3}^	0.480775^{5}^	0.546705^{8}^	0.453965^{4}^	0.610346^{9}^	0.4346^{2}^	0.329801^{1}^	0.507348^{6}^	0.518152^{7}^
	MRE	$\hat{\theta }$	0.314117^{8}^	0.280871^{2}^	0.307006^{7}^	0.300188^{5}^	0.306494^{6}^	0.320906^{10}^	0.280374^{1}^	0.317533^{9}^	0.284515^{3}^	0.290611^{4}^
		$\hat{k}$	1.149281^{9}^	1.024265^{4}^	1.096065^{7}^	1.114945^{8}^	1.070797^{6}^	1.170984^{10}^	1.017495^{3}^	0.978036^{1}^	1.007521^{2}^	1.062724^{5}^
	∑ *Ranks*		54^{9}^	16^{2}^	41^{8}^	39^{7}^	35^{6}^	58^{10}^	12^{1}^	27^{4}^	20^{3}^	28^{5}^
75	BIAS	$\hat{\theta }$	0.556078^{4}^	0.519778^{2}^	0.60888^{10}^	0.518784^{1}^	0.591903^{9}^	0.558229^{5}^	0.560574^{6}^	0.569369^{7}^	0.551783^{3}^	0.584388^{8}^
		$\hat{k}$	0.403045^{6}^	0.35918^{2}^	0.412658^{9}^	0.369824^{3}^	0.411775^{8}^	0.399558^{5}^	0.384624^{4}^	0.358486^{1}^	0.40391^{7}^	0.424945^{10}^
	MSE	$\hat{\theta }$	0.565313^{9}^	0.458867^{2}^	0.619327^{10}^	0.458732^{1}^	0.561494^{7}^	0.562231^{8}^	0.544169^{5}^	0.500013^{3}^	0.527083^{4}^	0.545224^{6}^
		$\hat{k}$	0.336121^{10}^	0.232448^{2}^	0.308566^{6}^	0.268282^{3}^	0.299903^{5}^	0.33546^{9}^	0.289046^{4}^	0.20885^{1}^	0.309353^{7}^	0.324589^{8}^
	MRE	$\hat{\theta }$	0.222431^{4}^	0.207911^{2}^	0.243552^{10}^	0.207513^{1}^	0.236761^{9}^	0.223291^{5}^	0.22423^{6}^	0.227747^{7}^	0.220713^{3}^	0.233755^{8}^
		$\hat{k}$	0.80609^{6}^	0.718359^{2}^	0.825316^{9}^	0.739647^{3}^	0.82355^{8}^	0.799115^{5}^	0.769248^{4}^	0.716972^{1}^	0.807821^{7}^	0.849891^{10}^
	∑ *Ranks*		39^{7}^	12^{1.5}^	54^{10}^	12^{1.5}^	46^{8}^	37^{6}^	29^{4}^	20^{3}^	31^{5}^	50^{9}^
150	BIAS	$\hat{\theta }$	0.390752^{2}^	0.381862^{1}^	0.436288^{8}^	0.393323^{3}^	0.437085^{9}^	0.4074^{6}^	0.396363^{4}^	0.405977^{5}^	0.446504^{10}^	0.413408^{7}^
		$\hat{k}$	0.287464^{4}^	0.257973^{2}^	0.291922^{6}^	0.28912^{5}^	0.292221^{7}^	0.295247^{8}^	0.272192^{3}^	0.248454^{1}^	0.314795^{10}^	0.304466^{9}^
	MSE	$\hat{\theta }$	0.307497^{6}^	0.265526^{2}^	0.351204^{9}^	0.284106^{4}^	0.326407^{7}^	0.327141^{8}^	0.274368^{3}^	0.265147^{1}^	0.365975^{10}^	0.300644^{5}^
		$\hat{k}$	0.18598^{7}^	0.136653^{2}^	0.168551^{5}^	0.179392^{6}^	0.163095^{4}^	0.192868^{9}^	0.153845^{3}^	0.111208^{1}^	0.211127^{10}^	0.18705^{8}^
	MRE	$\hat{\theta }$	0.156301^{2}^	0.152745^{1}^	0.174515^{8}^	0.157329^{3}^	0.174834^{9}^	0.16296^{6}^	0.158545^{4}^	0.162391^{5}^	0.178602^{10}^	0.165363^{7}^
		$\hat{k}$	0.574928^{4}^	0.515946^{2}^	0.583844^{6}^	0.57824^{5}^	0.584442^{7}^	0.590494^{8}^	0.544384^{3}^	0.496908^{1}^	0.629591^{10}^	0.608931^{9}^
	∑ *Ranks*		25^{4}^	10^{1}^	42^{6}^	26^{5}^	43^{7}^	45^{8.5}^	20^{3}^	14^{2}^	60^{10}^	45^{8.5}^
250	BIAS	$\hat{\theta }$	0.296113^{2}^	0.311227^{4}^	0.359134^{10}^	0.28001^{1}^	0.319913^{7}^	0.319055^{6}^	0.300894^{3}^	0.326473^{8}^	0.346762^{9}^	0.316054^{5}^
		$\hat{k}$	0.213243^{6}^	0.210497^{4}^	0.238266^{9}^	0.201708^{2}^	0.211796^{5}^	0.229271^{8}^	0.210054^{3}^	0.1979^{1}^	0.245552^{10}^	0.225831^{7}^
	MSE	$\hat{\theta }$	0.172252^{3}^	0.184871^{6}^	0.238182^{10}^	0.150503^{1}^	0.198151^{7}^	0.206104^{8}^	0.16652^{2}^	0.177777^{4}^	0.221995^{9}^	0.178976^{5}^
		$\hat{k}$	0.104535^{7}^	0.094161^{4}^	0.11585^{8}^	0.085464^{2}^	0.09921^{5}^	0.123182^{10}^	0.089833^{3}^	0.0733^{1}^	0.123172^{9}^	0.101242^{6}^
	MRE	$\hat{\theta }$	0.118445^{2}^	0.124491^{4}^	0.143654^{10}^	0.112004^{1}^	0.127965^{7}^	0.127622^{6}^	0.120358^{3}^	0.130589^{8}^	0.138705^{9}^	0.126422^{5}^
		$\hat{k}$	0.426486^{6}^	0.420994^{4}^	0.476532^{9}^	0.403416^{2}^	0.423591^{5}^	0.458542^{8}^	0.420108^{3}^	0.3958^{1}^	0.491105^{10}^	0.451662^{7}^
	∑ *Ranks*		26^{4.5}^	26^{4.5}^	56^{9.5}^	9^{1}^	36^{7}^	46^{8}^	17^{2}^	23^{3}^	56^{9.5}^	35^{6}^
400	BIAS	$\hat{\theta }$	0.234012^{4}^	0.220029^{2}^	0.267698^{9}^	0.212383^{1}^	0.253653^{6}^	0.232454^{3}^	0.241337^{5}^	0.276066^{10}^	0.266256^{8}^	0.256787^{7}^
		$\hat{k}$	0.161969^{4}^	0.148843^{2}^	0.167631^{8}^	0.148275^{1}^	0.162108^{5}^	0.159191^{3}^	0.166232^{7}^	0.162454^{6}^	0.186654^{10}^	0.184951^{9}^
	MSE	$\hat{\theta }$	0.103079^{4}^	0.086864^{2}^	0.136364^{9}^	0.086863^{1}^	0.113036^{6}^	0.093326^{3}^	0.109369^{5}^	0.130232^{8}^	0.13961^{10}^	0.117564^{7}^
		$\hat{k}$	0.055217^{6}^	0.041391^{1}^	0.060733^{8}^	0.045937^{2}^	0.050541^{5}^	0.049931^{4}^	0.056501^{7}^	0.048659^{3}^	0.078529^{10}^	0.067448^{9}^
	MRE	$\hat{\theta }$	0.093605^{4}^	0.088011^{2}^	0.107079^{9}^	0.084953^{1}^	0.101461^{6}^	0.092982^{3}^	0.096535^{5}^	0.110427^{10}^	0.106502^{8}^	0.102715^{7}^
		$\hat{k}$	0.323938^{4}^	0.297686^{2}^	0.335263^{8}^	0.29655^{1}^	0.324215^{5}^	0.318382^{3}^	0.332465^{7}^	0.324909^{6}^	0.373308^{10}^	0.369901^{9}^
	∑ *Ranks*		26^{4}^	11^{2}^	51^{9}^	7^{1}^	33^{5}^	19^{3}^	36^{6}^	43^{7}^	56^{10}^	48^{8}^
600	BIAS	$\hat{\theta }$	0.166568^{2}^	0.183395^{3}^	0.198672^{6}^	0.158528^{1}^	0.213303^{9}^	0.187724^{4}^	0.193838^{5}^	0.221978^{10}^	0.212191^{8}^	0.205696^{7}^
		$\hat{k}$	0.114521^{2}^	0.122356^{3}^	0.127214^{4}^	0.113139^{1}^	0.134436^{7}^	0.129248^{6}^	0.127249^{5}^	0.135604^{8}^	0.147815^{10}^	0.139708^{9}^
	MSE	$\hat{\theta }$	0.0513^{2}^	0.060536^{4}^	0.068831^{6}^	0.048841^{1}^	0.077211^{8}^	0.060108^{3}^	0.064467^{5}^	0.082234^{10}^	0.082038^{9}^	0.071342^{7}^
		$\hat{k}$	0.025609^{2}^	0.029524^{4}^	0.029326^{3}^	0.025118^{1}^	0.033026^{8}^	0.029978^{6}^	0.029689^{5}^	0.032099^{7}^	0.043367^{10}^	0.034564^{9}^
	MRE	$\hat{\theta }$	0.066627^{2}^	0.073358^{3}^	0.079469^{6}^	0.063411^{1}^	0.085321^{9}^	0.07509^{4}^	0.077535^{5}^	0.088791^{10}^	0.084877^{8}^	0.082279^{7}^
		$\hat{k}$	0.229043^{2}^	0.244712^{3}^	0.254428^{4}^	0.226277^{1}^	0.268873^{7}^	0.258497^{6}^	0.254499^{5}^	0.271209^{8}^	0.29563^{10}^	0.279416^{9}^
	∑ *Ranks*		12^{2}^	20^{3}^	29^{4.5}^	6^{1}^	48^{7.5}^	29^{4.5}^	30^{6}^	53^{9}^	55^{10}^	48^{7.5}^

**Table 4:** Simulation values of BIAS, MSE and MRE for (*θ* = 1.5, *k* = 2.5).

n	Est.	Est. Par.	MLE	ADE	CVME	MPSE	LSE	RTADE	WLSE	LTADE	MSADE	MSALDE
30	BIAS	$\hat{\theta }$	0.428237^{2}^	0.468979^{6}^	0.539906^{10}^	0.40148^{1}^	0.504608^{8}^	0.453541^{4}^	0.470275^{7}^	0.50586^{9}^	0.451722^{3}^	0.455486^{5}^
		$\hat{k}$	0.836256^{2}^	0.919593^{7}^	1.0129^{10}^	0.796292^{1}^	0.964762^{9}^	0.918323^{6}^	0.91693^{5}^	0.928209^{8}^	0.850541^{3}^	0.892521^{4}^
	MSE	$\hat{\theta }$	0.329386^{2}^	0.417309^{7}^	0.574808^{10}^	0.264389^{1}^	0.475389^{8}^	0.37871^{4}^	0.406551^{6}^	0.49733^{9}^	0.385381^{5}^	0.361543^{3}^
		$\hat{k}$	1.258945^{2}^	1.629591^{7}^	1.974094^{10}^	1.097963^{1}^	1.777325^{9}^	1.500292^{5}^	1.519597^{6}^	1.711998^{8}^	1.473868^{3}^	1.476971^{4}^
	MRE	$\hat{\theta }$	0.285491^{2}^	0.312653^{6}^	0.359937^{10}^	0.267654^{1}^	0.336405^{8}^	0.302361^{4}^	0.313516^{7}^	0.33724^{9}^	0.301148^{3}^	0.303658^{5}^
		$\hat{k}$	0.334502^{2}^	0.367837^{7}^	0.40516^{10}^	0.318517^{1}^	0.385905^{9}^	0.367329^{6}^	0.366772^{5}^	0.371284^{8}^	0.340216^{3}^	0.357008^{4}^
	∑ *Ranks*		12^{2}^	40^{7}^	60^{10}^	6^{1}^	51^{8.5}^	29^{5}^	36^{6}^	51^{8.5}^	20^{3}^	25^{4}^
75	BIAS	$\hat{\theta }$	0.240096^{1}^	0.277541^{4}^	0.311653^{9}^	0.240826^{2}^	0.28406^{6}^	0.25541^{3}^	0.282904^{5}^	0.313344^{10}^	0.290153^{7}^	0.291699^{8}^
		$\hat{k}$	0.459304^{2}^	0.520108^{5}^	0.584592^{10}^	0.450679^{1}^	0.517831^{4}^	0.50681^{3}^	0.538249^{7}^	0.566009^{9}^	0.532248^{6}^	0.560438^{8}^
	MSE	$\hat{\theta }$	0.098375^{2}^	0.128617^{4}^	0.180728^{10}^	0.094473^{1}^	0.139489^{8}^	0.113331^{3}^	0.136844^{6}^	0.167995^{9}^	0.13703^{7}^	0.134782^{5}^
		$\hat{k}$	0.354397^{2}^	0.475904^{4}^	0.633613^{10}^	0.336473^{1}^	0.478706^{5}^	0.437934^{3}^	0.493566^{7}^	0.549583^{9}^	0.487709^{6}^	0.528784^{8}^
	MRE	$\hat{\theta }$	0.160064^{1}^	0.185027^{4}^	0.207768^{9}^	0.16055^{2}^	0.189373^{6}^	0.170273^{3}^	0.188603^{5}^	0.208896^{10}^	0.193435^{7}^	0.194466^{8}^
		$\hat{k}$	0.183722^{2}^	0.208043^{5}^	0.233837^{10}^	0.180272^{1}^	0.207132^{4}^	0.202724^{3}^	0.2153^{7}^	0.226404^{9}^	0.212899^{6}^	0.224175^{8}^
	∑ *Ranks*		10^{2}^	26^{4}^	58^{10}^	8^{1}^	33^{5}^	18^{3}^	37^{6}^	56^{9}^	39^{7}^	45^{8}^
150	BIAS	$\hat{\theta }$	0.177541^{2}^	0.184896^{3}^	0.207501^{9}^	0.168576^{1}^	0.197421^{7}^	0.185099^{4}^	0.189747^{5}^	0.219683^{10}^	0.203734^{8}^	0.193494^{6}^
		$\hat{k}$	0.333253^{2}^	0.346778^{3}^	0.387429^{9}^	0.325864^{1}^	0.368483^{6}^	0.363104^{5}^	0.355326^{4}^	0.398618^{10}^	0.386283^{8}^	0.371089^{7}^
	MSE	$\hat{\theta }$	0.049545^{2}^	0.05462^{3}^	0.068953^{9}^	0.045328^{1}^	0.062977^{7}^	0.05501^{4}^	0.058104^{5}^	0.077923^{10}^	0.068784^{8}^	0.060082^{6}^
		$\hat{k}$	0.175698^{2}^	0.195385^{3}^	0.238082^{8}^	0.170771^{1}^	0.216293^{6}^	0.20996^{5}^	0.204845^{4}^	0.257172^{9}^	0.257381^{10}^	0.228335^{7}^
	MRE	$\hat{\theta }$	0.118361^{2}^	0.123264^{3}^	0.138334^{9}^	0.112384^{1}^	0.131614^{7}^	0.123399^{4}^	0.126498^{5}^	0.146456^{10}^	0.135822^{8}^	0.128996^{6}^
		$\hat{k}$	0.133301^{2}^	0.138711^{3}^	0.154971^{9}^	0.130346^{1}^	0.147393^{6}^	0.145242^{5}^	0.14213^{4}^	0.159447^{10}^	0.154513^{8}^	0.148435^{7}^
	∑ *Ranks*		12^{2}^	18^{3}^	53^{9}^	6^{1}^	39^{6.5}^	27^{4.5}^	27^{4.5}^	59^{10}^	50^{8}^	39^{6.5}^
250	BIAS	$\hat{\theta }$	0.121576^{1}^	0.141389^{5}^	0.155278^{7}^	0.127246^{2}^	0.156335^{8}^	0.133106^{3}^	0.139081^{4}^	0.175633^{10}^	0.160978^{9}^	0.149592^{6}^
		$\hat{k}$	0.232913^{1}^	0.271918^{5}^	0.285865^{6}^	0.239791^{2}^	0.290746^{7}^	0.253075^{3}^	0.270889^{4}^	0.324665^{10}^	0.309972^{9}^	0.291226^{8}^
	MSE	$\hat{\theta }$	0.024051^{1}^	0.031808^{5}^	0.038355^{8}^	0.025374^{2}^	0.037648^{7}^	0.027901^{3}^	0.031125^{4}^	0.049014^{10}^	0.042114^{9}^	0.03565^{6}^
		$\hat{k}$	0.089823^{1}^	0.117752^{5}^	0.129484^{6}^	0.093703^{2}^	0.132655^{7}^	0.100481^{3}^	0.115133^{4}^	0.164085^{10}^	0.160961^{9}^	0.13631^{8}^
	MRE	$\hat{\theta }$	0.081051^{1}^	0.094259^{5}^	0.103519^{7}^	0.08483^{2}^	0.104223^{8}^	0.088737^{3}^	0.092721^{4}^	0.117089^{10}^	0.107319^{9}^	0.099728^{6}^
		$\hat{k}$	0.093165^{1}^	0.108767^{5}^	0.114346^{6}^	0.095916^{2}^	0.116298^{7}^	0.10123^{3}^	0.108356^{4}^	0.129866^{10}^	0.123989^{9}^	0.116491^{8}^
	∑ *Ranks*		6^{1}^	30^{5}^	40^{6}^	12^{2}^	44^{8}^	18^{3}^	24^{4}^	60^{10}^	54^{9}^	42^{7}^
400	BIAS	$\hat{\theta }$	0.101447^{2}^	0.112216^{4}^	0.12131^{7}^	0.101365^{1}^	0.126729^{9}^	0.106828^{3}^	0.112363^{5}^	0.144215^{10}^	0.123133^{8}^	0.118446^{6}^
		$\hat{k}$	0.188667^{1}^	0.210178^{4}^	0.225908^{6}^	0.191616^{2}^	0.232767^{8}^	0.206085^{3}^	0.211991^{5}^	0.260573^{10}^	0.234827^{9}^	0.232116^{7}^
	MSE	$\hat{\theta }$	0.016077^{1}^	0.019936^{5}^	0.02274^{7}^	0.016187^{2}^	0.025459^{9}^	0.017955^{3}^	0.019566^{4}^	0.033104^{10}^	0.025196^{8}^	0.021645^{6}^
		$\hat{k}$	0.056805^{1}^	0.071034^{5}^	0.081055^{6}^	0.061311^{2}^	0.086655^{8}^	0.065993^{3}^	0.070714^{4}^	0.107024^{10}^	0.094386^{9}^	0.083321^{7}^
	MRE	$\hat{\theta }$	0.067631^{2}^	0.074811^{4}^	0.080874^{7}^	0.067576^{1}^	0.084486^{9}^	0.071218^{3}^	0.074908^{5}^	0.096144^{10}^	0.082089^{8}^	0.078964^{6}^
		$\hat{k}$	0.075467^{1}^	0.084071^{4}^	0.090363^{6}^	0.076647^{2}^	0.093107^{8}^	0.082434^{3}^	0.084796^{5}^	0.104229^{10}^	0.093931^{9}^	0.092846^{7}^
	∑ *Ranks*		8^{1}^	26^{4}^	39^{6.5}^	10^{2}^	51^{8.5}^	18^{3}^	28^{5}^	60^{10}^	51^{8.5}^	39^{6.5}^
600	BIAS	$\hat{\theta }$	0.084478^{2}^	0.091525^{4}^	0.099138^{7}^	0.079939^{1}^	0.101824^{8}^	0.08876^{3}^	0.092084^{5}^	0.111732^{10}^	0.107069^{9}^	0.098156^{6}^
		$\hat{k}$	0.161131^{2}^	0.173239^{4}^	0.18182^{6}^	0.147812^{1}^	0.186362^{8}^	0.170724^{3}^	0.176184^{5}^	0.20421^{10}^	0.198161^{9}^	0.185851^{7}^
	MSE	$\hat{\theta }$	0.011184^{2}^	0.012988^{4}^	0.015852^{7}^	0.010613^{1}^	0.016133^{8}^	0.012471^{3}^	0.013318^{5}^	0.019307^{10}^	0.018645^{9}^	0.014893^{6}^
		$\hat{k}$	0.040343^{2}^	0.047248^{4}^	0.05362^{6}^	0.040004^{1}^	0.054855^{8}^	0.045524^{3}^	0.048325^{5}^	0.065824^{10}^	0.065488^{9}^	0.053968^{7}^
	MRE	$\hat{\theta }$	0.056319^{2}^	0.061017^{4}^	0.066092^{7}^	0.053293^{1}^	0.067883^{8}^	0.059174^{3}^	0.061389^{5}^	0.074488^{10}^	0.071379^{9}^	0.065438^{6}^
		$\hat{k}$	0.064452^{2}^	0.069296^{4}^	0.072728^{6}^	0.059125^{1}^	0.074545^{8}^	0.06829^{3}^	0.070474^{5}^	0.081684^{10}^	0.079265^{9}^	0.07434^{7}^
	∑ *Ranks*		12^{2}^	24^{4}^	39^{6.5}^	6^{1}^	48^{8}^	18^{3}^	30^{5}^	60^{10}^	54^{9}^	39^{6.5}^

**Table 5:** Simulation values of BIAS, MSE and MRE for (*θ* = 2, *k* = 4).

n	Est.	Est. Par.	MLE	ADE	CVME	MPSE	LSE	RTADE	WLSE	LTADE	MSADE	MSALDE
30	BIAS	$\hat{\theta }$	0.459971^{2}^	0.485465^{4}^	0.524415^{9}^	0.42938^{1}^	0.521039^{8}^	0.491798^{6}^	0.487286^{5}^	0.574341^{10}^	0.495376^{7}^	0.478459^{3}^
		$\hat{k}$	0.978785^{2}^	1.031955^{6}^	1.131802^{9}^	0.89577^{1}^	1.086949^{8}^	1.073841^{7}^	1.027919^{5}^	1.180397^{10}^	1.017501^{3}^	1.022636^{4}^
	MSE	$\hat{\theta }$	0.391631^{2}^	0.400937^{4}^	0.656676^{10}^	0.31071^{1}^	0.587079^{8}^	0.518248^{6}^	0.405042^{5}^	0.634182^{9}^	0.550358^{7}^	0.397429^{3}^
		$\hat{k}$	1.812973^{2}^	1.83889^{3}^	3.110733^{10}^	1.370084^{1}^	2.647348^{8}^	2.585027^{7}^	1.846273^{4}^	2.740106^{9}^	2.508123^{6}^	1.847727^{5}^
	MRE	$\hat{\theta }$	0.229985^{2}^	0.242732^{4}^	0.262208^{9}^	0.21469^{1}^	0.260519^{8}^	0.245899^{6}^	0.243643^{5}^	0.287171^{10}^	0.247688^{7}^	0.239229^{3}^
		$\hat{k}$	0.244696^{2}^	0.257989^{6}^	0.282951^{9}^	0.223943^{1}^	0.271737^{8}^	0.26846^{7}^	0.25698^{5}^	0.295099^{10}^	0.254375^{3}^	0.255659^{4}^
	∑ *Ranks*		12^{2}^	27^{4}^	56^{9}^	6^{1}^	48^{8}^	39^{7}^	29^{5}^	58^{10}^	33^{6}^	22^{3}^
75	BIAS	$\hat{\theta }$	0.275412^{2}^	0.279612^{3}^	0.318161^{9}^	0.268217^{1}^	0.297231^{6}^	0.289344^{5}^	0.28255^{4}^	0.328143^{10}^	0.299918^{7}^	0.30761^{8}^
		$\hat{k}$	0.579713^{2}^	0.598982^{4}^	0.664116^{10}^	0.550663^{1}^	0.619428^{6}^	0.631339^{7}^	0.589579^{3}^	0.660948^{9}^	0.605344^{5}^	0.632185^{8}^
	MSE	$\hat{\theta }$	0.124369^{2}^	0.127425^{3}^	0.182594^{9}^	0.111594^{1}^	0.147896^{6}^	0.141173^{5}^	0.128689^{4}^	0.184212^{10}^	0.159792^{8}^	0.157292^{7}^
		$\hat{k}$	0.541313^{2}^	0.586614^{4}^	0.785648^{10}^	0.486109^{1}^	0.646668^{5}^	0.669927^{7}^	0.557997^{3}^	0.74147^{9}^	0.685115^{8}^	0.667484^{6}^
	MRE	$\hat{\theta }$	0.137706^{2}^	0.139806^{3}^	0.15908^{9}^	0.134109^{1}^	0.148615^{6}^	0.144672^{5}^	0.141275^{4}^	0.164072^{10}^	0.149959^{7}^	0.153805^{8}^
		$\hat{k}$	0.144928^{2}^	0.149746^{4}^	0.166029^{10}^	0.137666^{1}^	0.154857^{6}^	0.157835^{7}^	0.147395^{3}^	0.165237^{9}^	0.151336^{5}^	0.158046^{8}^
	∑ *Ranks*		12^{2}^	21^{3.5}^	57^{9.5}^	6^{1}^	35^{5}^	36^{6}^	21^{3.5}^	57^{9.5}^	40^{7}^	45^{8}^
150	BIAS	$\hat{\theta }$	0.191516^{3}^	0.190141^{2}^	0.218524^{7}^	0.184022^{1}^	0.219199^{8}^	0.192552^{4}^	0.204704^{5}^	0.230212^{10}^	0.224127^{9}^	0.214076^{6}^
		$\hat{k}$	0.410143^{3}^	0.403521^{2}^	0.451712^{6}^	0.383526^{1}^	0.456219^{8}^	0.413334^{4}^	0.432688^{5}^	0.463585^{9}^	0.466868^{10}^	0.454041^{7}^
	MSE	$\hat{\theta }$	0.058023^{3}^	0.057998^{2}^	0.079298^{8}^	0.053339^{1}^	0.076448^{7}^	0.061498^{4}^	0.067039^{5}^	0.08718^{9}^	0.087626^{10}^	0.069524^{6}^
		$\hat{k}$	0.268249^{3}^	0.262025^{2}^	0.33985^{8}^	0.235914^{1}^	0.330437^{7}^	0.284945^{4}^	0.295619^{5}^	0.35736^{9}^	0.388021^{10}^	0.315811^{6}^
	MRE	$\hat{\theta }$	0.095758^{3}^	0.09507^{2}^	0.109262^{7}^	0.092011^{1}^	0.1096^{8}^	0.096276^{4}^	0.102352^{5}^	0.115106^{10}^	0.112064^{9}^	0.107038^{6}^
		$\hat{k}$	0.102536^{3}^	0.10088^{2}^	0.112928^{6}^	0.095881^{1}^	0.114055^{8}^	0.103333^{4}^	0.108172^{5}^	0.115896^{9}^	0.116717^{10}^	0.11351^{7}^
	∑ *Ranks*		18^{3}^	12^{2}^	42^{7}^	6^{1}^	46^{8}^	24^{4}^	30^{5}^	56^{9}^	58^{10}^	38^{6}^
250	BIAS	$\hat{\theta }$	0.139547^{1}^	0.14858^{3}^	0.163836^{8}^	0.142465^{2}^	0.162957^{7}^	0.156059^{5}^	0.153998^{4}^	0.184722^{10}^	0.171493^{9}^	0.161637^{6}^
		$\hat{k}$	0.302751^{2}^	0.319038^{3}^	0.338714^{5}^	0.293552^{1}^	0.343119^{7}^	0.340899^{6}^	0.331344^{4}^	0.375904^{10}^	0.367654^{9}^	0.343432^{8}^
	MSE	$\hat{\theta }$	0.031915^{1}^	0.035888^{3}^	0.04377^{8}^	0.032676^{2}^	0.041025^{6}^	0.040967^{5}^	0.038567^{4}^	0.053771^{10}^	0.04811^{9}^	0.04152^{7}^
		$\hat{k}$	0.148014^{2}^	0.164322^{3}^	0.181996^{6}^	0.145345^{1}^	0.181463^{5}^	0.193599^{8}^	0.177473^{4}^	0.224925^{9}^	0.228541^{10}^	0.18821^{7}^
	MRE	$\hat{\theta }$	0.069774^{1}^	0.07429^{3}^	0.081918^{8}^	0.071233^{2}^	0.081478^{7}^	0.07803^{5}^	0.076999^{4}^	0.092361^{10}^	0.085746^{9}^	0.080819^{6}^
		$\hat{k}$	0.075688^{2}^	0.07976^{3}^	0.084679^{5}^	0.073388^{1}^	0.08578^{7}^	0.085225^{6}^	0.082836^{4}^	0.093976^{10}^	0.091914^{9}^	0.085858^{8}^
	∑ *Ranks*		9^{1.5}^	18^{3}^	40^{7}^	9^{1.5}^	39^{6}^	35^{5}^	24^{4}^	59^{10}^	55^{9}^	42^{8}^
400	BIAS	$\hat{\theta }$	0.108966^{2}^	0.123066^{4}^	0.128765^{7}^	0.107391^{1}^	0.125274^{5}^	0.127348^{6}^	0.115028^{3}^	0.141685^{10}^	0.139307^{9}^	0.132528^{8}^
		$\hat{k}$	0.23288^{2}^	0.260735^{5}^	0.272485^{7}^	0.218127^{1}^	0.258153^{4}^	0.268079^{6}^	0.248024^{3}^	0.288884^{9}^	0.292909^{10}^	0.281213^{8}^
	MSE	$\hat{\theta }$	0.01849^{1}^	0.023834^{4}^	0.026404^{7}^	0.018526^{2}^	0.024102^{5}^	0.02566^{6}^	0.021396^{3}^	0.03159^{10}^	0.031324^{9}^	0.02643^{8}^
		$\hat{k}$	0.084411^{2}^	0.107339^{5}^	0.117576^{7}^	0.084199^{1}^	0.101937^{4}^	0.115921^{6}^	0.097643^{3}^	0.130756^{9}^	0.14281^{10}^	0.118757^{8}^
	MRE	$\hat{\theta }$	0.054483^{2}^	0.061533^{4}^	0.064382^{7}^	0.053696^{1}^	0.062637^{5}^	0.063674^{6}^	0.057514^{3}^	0.070842^{10}^	0.069653^{9}^	0.066264^{8}^
		$\hat{k}$	0.05822^{2}^	0.065184^{5}^	0.068121^{7}^	0.054532^{1}^	0.064538^{4}^	0.06702^{6}^	0.062006^{3}^	0.072221^{9}^	0.073227^{10}^	0.070303^{8}^
	∑ *Ranks*		11^{2}^	27^{4.5}^	42^{7}^	7^{1}^	27^{4.5}^	36^{6}^	18^{3}^	57^{9.5}^	57^{9.5}^	48^{8}^
600	BIAS	$\hat{\theta }$	0.088926^{2}^	0.099215^{5}^	0.105833^{8}^	0.084026^{1}^	0.105394^{7}^	0.096079^{3}^	0.098024^{4}^	0.11408^{9}^	0.115342^{10}^	0.104096^{6}^
		$\hat{k}$	0.190161^{2}^	0.207523^{4}^	0.219344^{7}^	0.164448^{1}^	0.217247^{6}^	0.206334^{3}^	0.211948^{5}^	0.235627^{9}^	0.245342^{10}^	0.224583^{8}^
	MSE	$\hat{\theta }$	0.012523^{2}^	0.01544^{5}^	0.01843^{8}^	0.011765^{1}^	0.017558^{6}^	0.015087^{4}^	0.014736^{3}^	0.020524^{9}^	0.021373^{10}^	0.017563^{7}^
		$\hat{k}$	0.058129^{2}^	0.069122^{4}^	0.07915^{7}^	0.052886^{1}^	0.075307^{6}^	0.069643^{5}^	0.068381^{3}^	0.086258^{9}^	0.0986^{10}^	0.080964^{8}^
	MRE	$\hat{\theta }$	0.044463^{2}^	0.049608^{5}^	0.052916^{8}^	0.042013^{1}^	0.052697^{7}^	0.048039^{3}^	0.049012^{4}^	0.05704^{9}^	0.057671^{10}^	0.052048^{6}^
		$\hat{k}$	0.04754^{2}^	0.051881^{4}^	0.054836^{7}^	0.041112^{1}^	0.054312^{6}^	0.051583^{3}^	0.052987^{5}^	0.058907^{9}^	0.061336^{10}^	0.056146^{8}^
	∑ *Ranks*		12^{2}^	27^{5}^	45^{8}^	6^{1}^	38^{6}^	21^{3}^	24^{4}^	54^{9}^	60^{10}^	43^{7}^

**Table 6:** Partial and overall ranks of all the methods of estimation of GD by various values of model parameters.

Parameter	*n*	MLE	ADE	CVME	MPSE	LSE	RTADE	WLSE	LTADE	MSADE	MSALDE
*θ* = 0.25, *k* = 0.5	30	7.0	4.0	10.0	3.0	9.0	6.0	5.0	8.0	2.0	1.0
	75	5.5	7.0	10.0	1.0	8.0	5.5	4.0	9.0	3.0	2.0
	150	4.0	6.0	10.0	1.0	7.0	8.0	5.0	9.0	3.0	2.0
	250	2.0	5.0	10.0	1.0	9.0	3.0	7.0	8.0	6.0	4.0
	400	6.0	5.0	10.0	1.0	7.0	4.0	2.0	9.0	8.0	3.0
	600	3.0	4.0	8.0	1.0	9.5	6.0	2.0	9.5	7.0	5.0
*θ* = 0.5, *k* = 1.5	30	3.0	5.5	8.5	5.5	10.0	4.0	8.5	7.0	1.0	2.0
	75	1.0	4.0	9.5	5.0	9.5	2.0	8.0	7.0	3.0	6.0
	150	1.0	4.5	10.0	3.0	9.0	2.0	6.0	8.0	4.5	7.0
	250	1.0	4.0	8.0	3.0	9.0	2.0	5.0	10.0	7.0	6.0
	400	1.0	4.0	8.0	3.0	9.5	2.0	5.0	9.5	7.0	6.0
	600	1.0	4.0	10.0	2.0	8.0	3.0	5.0	9.0	6.0	7.0
*θ* = 2.5, *k* = 0.5	30	9.0	2.0	8.0	7.0	6.0	10.0	1.0	4.0	3.0	5.0
	75	7.0	1.5	10.0	1.5	8.0	6.0	4.0	3.0	5.0	9.0
	150	4.0	1.0	6.0	5.0	7.0	8.5	3.0	2.0	10.0	8.5
	250	4.5	4.5	9.5	1.0	7.0	8.0	2.0	3.0	9.5	6.0
	400	4.0	2.0	9.0	1.0	5.0	3.0	6.0	7.0	10.0	8.0
	600	2.0	3.0	4.5	1.0	7.5	4.5	6.0	9.0	10.0	7.5
*θ* = 1.5, *k* = 2.5	30	2.0	7.0	10.0	1.0	8.5	5.0	6.0	8.5	3.0	4.0
	75	2.0	4.0	10.0	1.0	5.0	3.0	6.0	9.0	7.0	8.0
	150	2.0	3.0	9.0	1.0	6.5	4.5	4.5	10.0	8.0	6.5
	250	1.0	5.0	6.0	2.0	8.0	3.0	4.0	10.0	9.0	7.0
	400	1.0	4.0	6.5	2.0	8.5	3.0	5.0	10.0	8.5	6.5
	600	2.0	4.0	6.5	1.0	8.0	3.0	5.0	10.0	9.0	6.5
*θ* = 2, *k* = 4	30	2.0	4.0	9.0	1.0	8.0	7.0	5.0	10.0	6.0	3.0
	75	2.0	3.5	9.5	1.0	5.0	6.0	3.5	9.5	7.0	8.0
	150	3.0	2.0	7.0	1.0	8.0	4.0	5.0	9.0	10.0	6.0
	250	1.5	3.0	7.0	1.5	6.0	5.0	4.0	10.0	9.0	8.0
	400	2.0	4.5	7.0	1.0	4.5	6.0	3.0	9.5	9.5	8.0
	600	2.0	5.0	8.0	1.0	6.0	3.0	4.0	9.0	10.0	7.0
∑ Ranks		88.5	120.0	254.5	60.5	227.0	140.0	139.5	245.5	201.0	173.5
Overall Rank		2	3	10	1	8	5	4	9	7	6

## 7 Real data analysis

The flexibility of the distribution is shown in this section via the use of data taken from the real world. The evaluated data is a COVID-19 data set of 30 days of mortality rate that belonged to the Netherlands and was captured from the 31st of March to the 30th of April 2020. It was based on the death rate in the general population, and it is available at https://covid19.who.int/.

In order to illustrate how flexible GD is, we shall evaluate it in comparison to a number of well-known models, such as exponential distribution (ED), Frechet distribution (FD), Lindley distribution (LD), modified Kies exponential distribution (MKED) [19], Lomax distribution (L0D), Weibull Frechet distribution (WFD) [20], Frechet Weibull distribution (FWD) [21], Burr-Hatke distribution (BHD) [22], inverse log-logistic distribution (ILLD) [23], inversely weighted Lindley distribution (IWLD) [24], type I generalized half logistic distribution (TIGHLD) [25], half-logistic distribution (HLD) and Maxwell distribution (MD).

In order to determine which is the most appropriate model to use with the COVID-19 data set, we make use of a number of analytical criteria, among which are: the Akaike information criterion (*Cr*_1_), the correct Akaike information criterion (*Cr*_2_), Bayesian information criterion (*Cr*_3_), Hannan information criterion (*Cr*_4_). In addition to this, we base our choice on a variety of additional data about the model’s overall goodness-of-fit, such as Anderson Darling (*G*_1_), Cramér–von Mises (*G*_2_) and Kolmogorov–Smirnov (*G*_3_) with its p-value (*G*_3_(*p*)). The model with the minimum values of these measures is the best model for fitting the COVID-19 data set.

**Fig 3:**
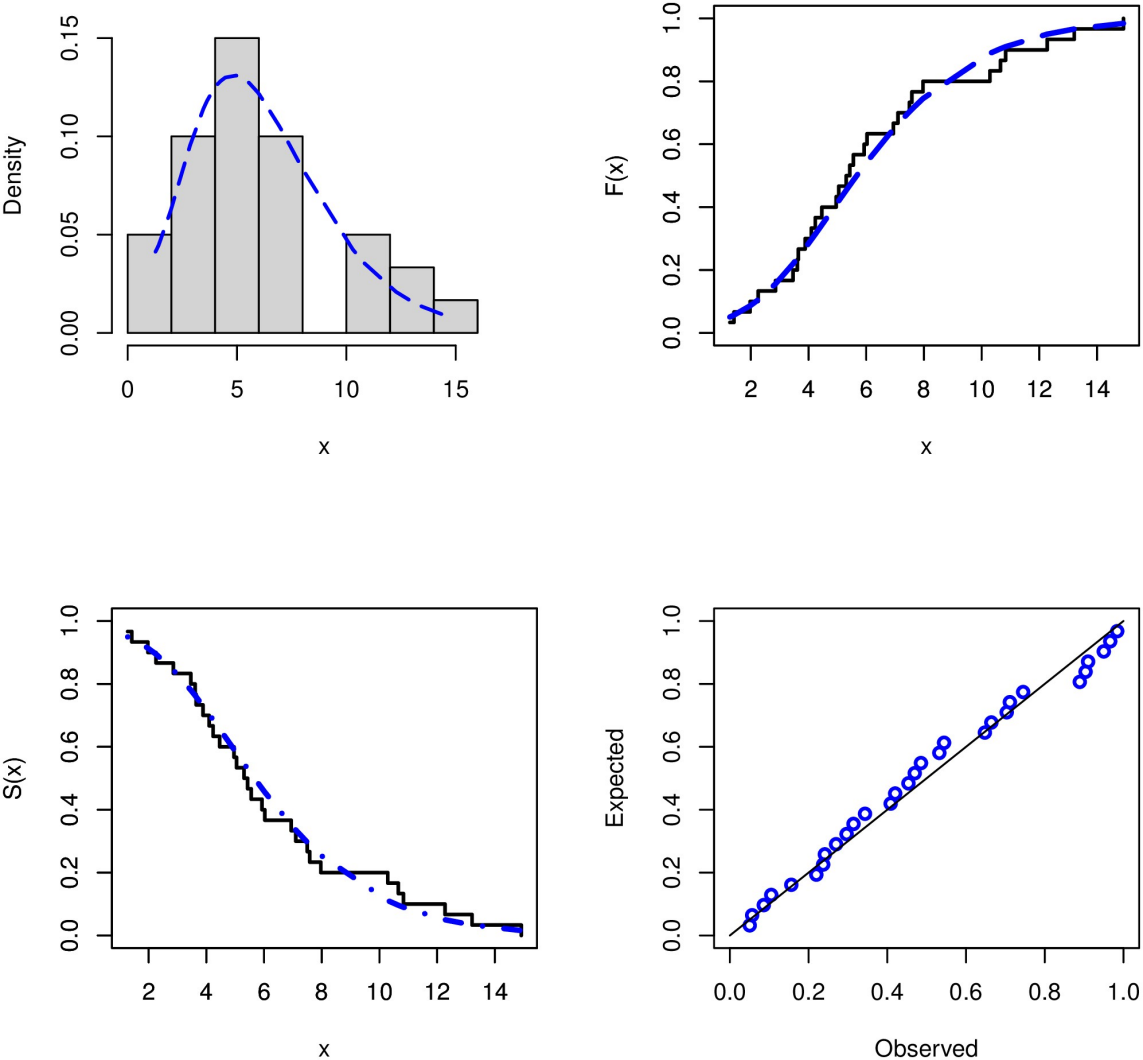
Histogram of the COVID-19 data set with the fitted PDF, CDF, SF, and P-P plots.

**Fig 4:**
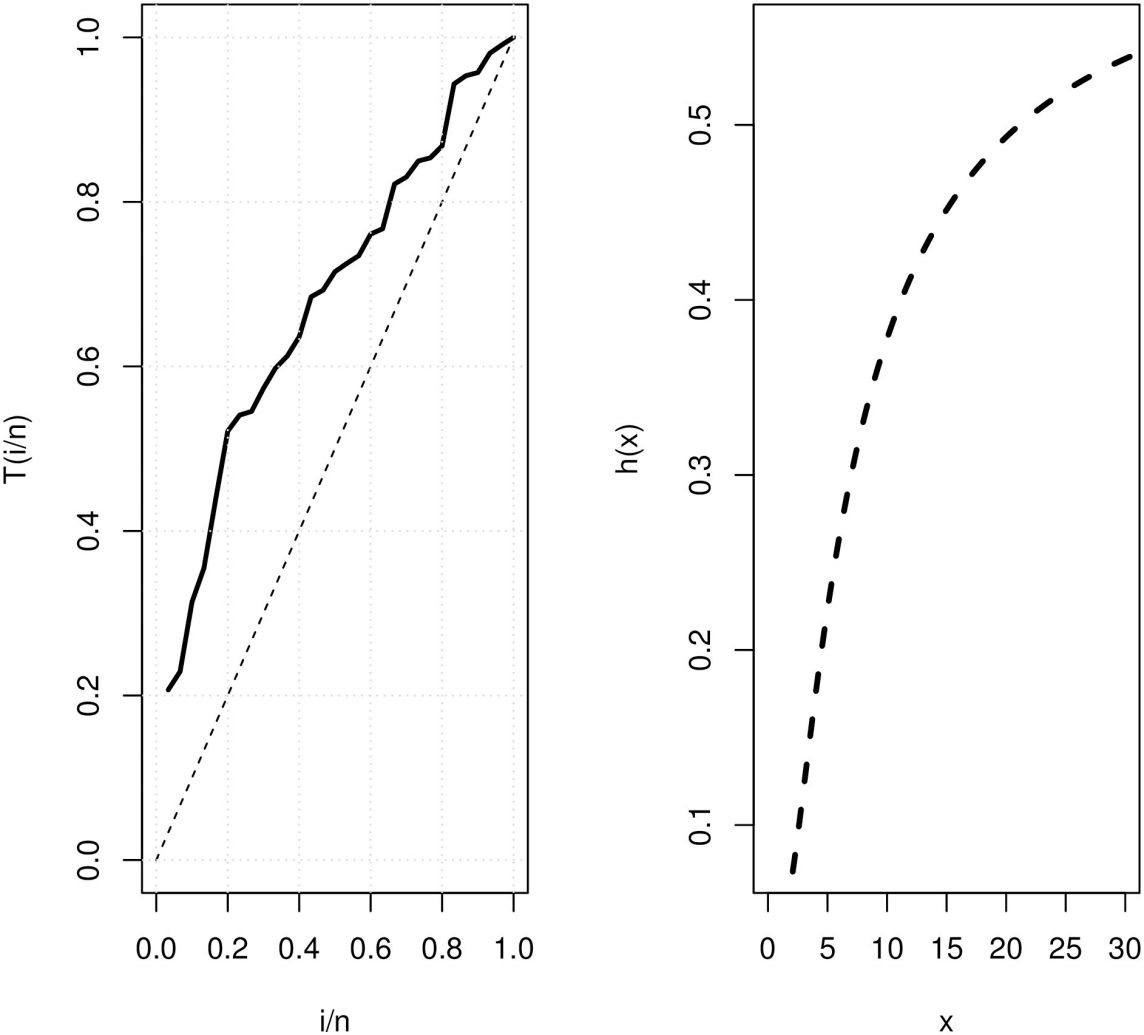
TTT plot and fitted HRF of GD for the COVID-19 data set.

**Fig 5:**
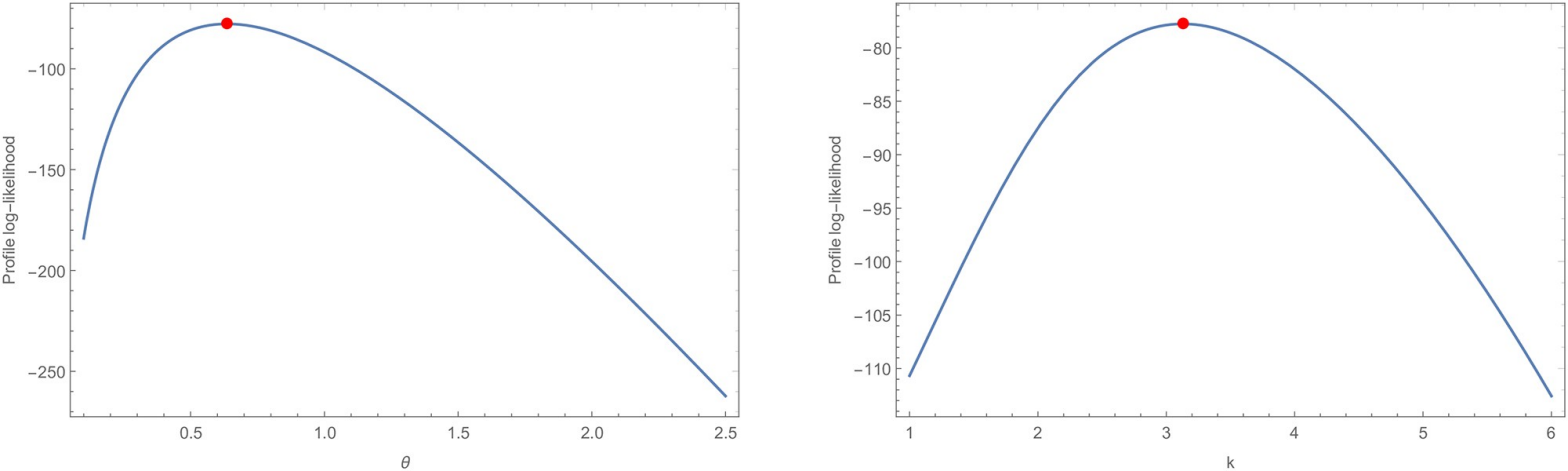
The profile of the log-likelihood functions for *θ* and *k* parameters of the COVID-19 data set.

Analytical measurements, as well as the estimates by MLE and corresponding standard errors (SE), are supplied for the COVID-19 data set that was being considered for evaluation. These numerical values are reported in Table 7, as shown. As a consequence, the GD performs better than the other models that are equivalent to it. The P-P plot and the fitted PDF, CDF, and SF plots are used to fit GD to the COVID-19 data set, which is shown in Fig 3. Using the COVID-19 data set, the GD was shown to be a good fit. TTT and estimated HRF of GD plots are shown in Fig 4 for the COVID-19 data set. The behavior of the log-likelihood function with estimated parameters is shown in Fig 5 for the COVID-19 data set, which is a unimodal function.

**Table 7:** Numerical values for analyzing the COVID-19 real data set.

Model	−L	*Cr*_1_	*Cr*_2_	*Cr*_3_	*Cr*_4_	*G*_1_	*G*_2_	*G*_3_	*G*_3_(*p*)	Est. parameters (SEs)
GD	77.7688	159.538	159.982	162.34	160.434	0.284572	0.0392159	0.0891151	0.971048	$\hat{\theta }=0.636297\;\left(0.121347\right)$
										$\hat{k}=3.12766\;\left(0.566639\right)$
ED	84.5253	171.051	171.193	172.452	171.499	2.70761	0.500305	0.263364	0.0311642	$\hat{a}=0.162431\;\left(0.0296556\right)$
FD	81.037	166.074	166.518	168.876	166.971	0.930476	0.14033	0.151265	0.498527	$\hat{a}=3.77888\;\left(0.473233\right)$
										$\hat{b}=1.55012\;\left(0.201445\right)$
LD	79.964	161.928	162.071	163.329	162.376	1.06265	0.170447	0.17938	0.28923	$\hat{\alpha }=0.288492\;\left(0.0377199\right)$
MKED	77.9252	159.85	160.295	162.653	160.747	0.514015	0.09486	0.132623	0.666945	$\hat{\alpha }=1.36564\;\left(0.20207\right)$
										$\hat{\lambda }=0.0943393\;\left(0.00922255\right)$
LoD	84.5253	173.051	173.495	175.853	173.947	2.70783	0.500364	0.263377	0.0311514	$\hat{\lambda }=1.48247\times 10^{8}\;\left(5.28653\times 10^{11}\right)$
										$\hat{\beta }=9.1264\times 10^{8}\;\left(3.25436\times 10^{12}\right)$
WFD	112.875	233.749	235.349	239.354	235.542	0.806709	0.116411	0.130224	0.68905	$\hat{\alpha }=101.075\;\left(49.5191\right)$
										$\hat{\beta }=1.03422\;\left(0.150336\right)$
										$\hat{a}=4.58572\;\left(1.61262\right)$
										$\hat{b}=0.0798205\;\left(0.046444\right)$
FWD	81.037	170.074	171.674	175.679	171.867	0.930476	0.14033	0.151265	0.498527	$\hat{\alpha }=1.38783\;\left(9.3682\right)$
										$\hat{\beta }=1.88815\;\left(6.00894\right)$
										$\hat{k}=1.11694\;\left(7.53968\right)$
										$\hat{\lambda }=2.13907\;\left(5.99898\right)$
BHD	110.751	223.501	223.644	224.902	223.95	23.1985	4.67369	0.623323	<0.00001	$\hat{a}=0.0188456\;\left(0.0273239\right)$
ILLD	110.764	223.528	223.671	224.929	223.976	19.0442	4.03571	0.587968	<0.00001	$\hat{\alpha }=0.904898\;\left(0.129895\right)$
IWLD	79.3334	162.667	163.111	165.469	163.563	0.647407	0.0943952	0.125948	0.728068	$\hat{\alpha }=2.61509\;\left(0.643228\right)$
										$\hat{\lambda }=11.8725\;\left(3.01831\right)$
TIGHLD	82.0426	166.085	166.228	167.486	166.533	1.92595	0.346689	0.23247	0.078125	$\hat{a}=0.182004\;\left(0.0332293\right)$
HLD	81.4583	164.917	165.059	166.318	165.365	1.74633	0.309252	0.221941	0.104093	$\hat{b}=0.237018\;\left(0.0350458\right)$
MD	79.524	161.048	161.191	162.449	161.496	1.72138	0.26361	0.170229	0.349596	$\hat{\alpha }=0.0300176\;\left(0.00447476\right)$

## 8 Conclusion

In this paper, we derived a general statistical model using a mix of exponential and gamma distributions called a general two-parameter distribution. The formulation of the PDF of the general model was derived in detail with its CDF and HRF. The behavior of PDF and HRF of GTPD at points *x* = 0 and *x* = ∞ were calculated. Also, the shapes of both PDF and HRF of GTPD were determined mathematically. Many statistical properties of the GTPD were determined, such as moments with its related measures, incomplete moments with its related measures, entropy, and stochastic orders. Ten different estimation methods were used to calculate unknown parameters of the GTPD. Gemeay distribution was presented as a special case of the GTPD. The randomly generated data sets from GD were used to check the performance of the different estimation methods. The flexibility of GD was illustrated by using mortality rate of the COVID-19 real data set, which showed that GD is the best model for fitting the analyzed COVID-19 data set than other compared well-known models.
